# Antiplasmodial and Cytotoxic Cytochalasins from an Endophytic Fungus, *Nemania* sp. UM10M, Isolated from a Diseased *Torreya taxifolia* Leaf

**DOI:** 10.3390/molecules24040777

**Published:** 2019-02-21

**Authors:** Mallika Kumarihamy, Daneel Ferreira, Edward M. Croom, Rajnish Sahu, Babu L. Tekwani, Stephen O. Duke, Shabana Khan, Natascha Techen, N. P. Dhammika Nanayakkara

**Affiliations:** 1 National Center for Natural Products Research, Research Institute of Pharmaceutical Sciences, University of Mississippi, University, MS 38677, USA; mkumarih@olemiss.edu (M.K.); rsahu@alasu.edu (R.S.); btekwani@southenresearch.org (B.L.T.); skhan@olemiss.edu (S.K.); ntechen@olemiss.edu (N.T.); 2 Department of BioMolecular Sciences, Division of Pharmacognosy, School of Pharmacy, The University of Mississippi, University, MS 38677, USA; dferreir@olemiss.edu (D.F.); emcroom@olemiss.edu (E.M.C.J.); 3 Natural Products Utilization Research Unit, USDA-ARS, University, MS 38677, USA; stephen.duke@ars.usda.gov

**Keywords:** *Nemania*, Xylariaceae, *Torreya taxifolia*, plant pathogenic and endophytic fungi, cytochalasins, malaria, cytotoxicity, phytotoxicity

## Abstract

Bioassay-guided fractionation of an EtOAc extract of the broth of the endophytic fungus *Nemania* sp. UM10M (Xylariaceae) isolated from a diseased *Torreya taxifolia* leaf afforded three known cytochalasins, 19,20-epoxycytochalasins C (**1**) and D (**2**), and 18-deoxy-19,20-epoxy-cytochalasin C (**3**). All three compounds showed potent in vitro antiplasmodial activity and phytotoxicity with no cytotoxicity to Vero cells. These compounds exhibited moderate to weak cytotoxicity to some of the cell lines of a panel of solid tumor (SK-MEL, KB, BT-549, and SK-OV-3) and kidney epithelial cells (LLC-PK_11_). Evaluation of in vivo antimalarial activity of 19,20-epoxycytochalasin C (**1**) in a mouse model at 100 mg/kg dose showed that this compound had weak suppressive antiplasmodial activity and was toxic to animals.

## 1. Introduction

Malaria remains a serious threat to human health in many parts of the tropics and subtropics, and an estimated 216 million cases and 445,000 deaths occurred worldwide in 2016 [1]. This disease is caused by apicomplexan parasites of the genus *Plasmodium*. *P. falciparum*, which is responsible for the majority of malaria deaths, has developed resistance to all currently available drugs, and mosquito vectors have become resistant to one or more insecticides in all WHO regions [1]. There is an urgent need to develop novel drugs with new modes of action for effective control of this disease.

Apicomplexan parasites such as *Plasmodia* contain the apicoplast organelle that is similar to plastids found in the cells of photosynthetic organisms with the same genetic complements [2]. Apicoplasts have some essential plant-like metabolic pathways which are absent in mammalian hosts. Over the last decade, a number of validated targets in plant-like metabolic pathways in the apicoplast of malaria parasites have been reported [3,4,5,6,7]. Apicoplasts perform vital metabolic functions and are essential for both erythrocytic and hepatic development of the parasites in mammalian hosts [3,4,5,6,7]. Therefore, plant-like metabolic pathways in apicoplasts serve as promising targets for antimalarial drug discovery [3,4,5,6,7].

Most phytotoxins disrupt metabolic pathways in plants, including those in plastids [8]. Several studies have been carried out to evaluate the in vitro antiplasmodial activity of herbicides and phytotoxins that act on known apicoplast metabolic pathways [9,10,11], and some of them have shown activity against *Plasmodium*.

**Figure 1:**
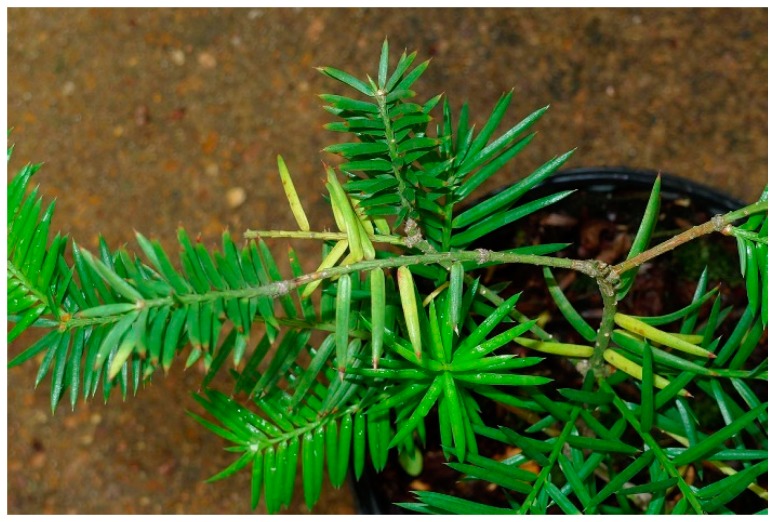
Symptoms of diseased needles of cultivated *T. taxifolia*.

We have screened a number of plant pathogenic fungal extracts with phytotoxic activity for their antiplasmodial activity. Some of these extracts yielded compounds with good antiplasmodial activity [12,13,14]. As part of this program, we investigated endophytic fungi isolated from a diseased leaf of cultivated *Torreya taxifolia* Arnott (Figure 1).

**Figure 2:**
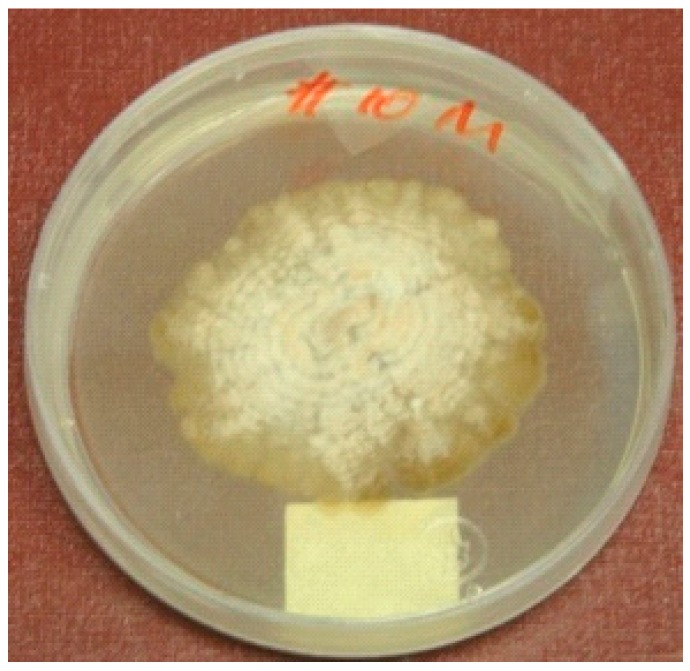
Potato dextrose agar plate of the fungus UM10M.

One of the fungal extracts showed potent phytotoxic activity and selective antiplasmodial activity. This fungus (UM10M) (Figure 2) was identified as a species of the genus *Nemania* (Xylariaceae). Some members of this family have been identified as endophytes and plant pathogens [15].

**Figure 3:**
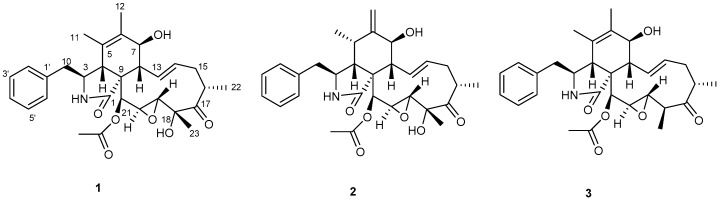
Structures of compounds **1**–**3** isolated from the fungus UM10M.

Bioassay guided fractionation of this extract led to the isolation of three cytochalasins, 19,20-epoxycytochalasins C (**1**) and D (**2**), and 18-deoxy-19,20-epoxycytochalasin C (**3**) (Figure 3), as the compounds responsible for both the phytotoxic and antimalarial activity.

A number of cytochalasins have been reported from Xylariaceae and several other plant pathogenic and endophytic ascomycete and basidiomycete genera (16). Cytochalasins have shown diverse biological activities including antiplasmodial [16,17], antitoxoplasma [18], and phytotoxic [19,20,21] activities. Cytochalasin B, which is known to inhibit actin filament formation, is capable of interfering with several cellular processes including cytokinesis, intracellular motility, and exo and endocytosis [22,23]. This compound is extensively used to study cytoskeletal mechanisms [22,23].

Compounds **1** and **2** showed potent antimalarial activity against both chloroquine-sensitive and -resistant clones and were not cytotoxic to Vero cells at the highest concentration tested (4760 ng/mL) whereas compound **3** showed moderate activity. Compound **1** which was isolated as the major compound was evaluated for in vivo antimalarial activity in a mouse model.

## 2. Results and Discussion

An EtOAc extract of the fermentation broth of the endophytic fungus isolated from a diseased *T. taxifolia* leaf showed good phytotoxic activity against both a dicot (lettuce, *Lactuca sativa* L.) and a monocot (bentgrass, *Agrostis stolonifera* L.) and potent antiplasmodial activity against chloroquine-sensitive (D6) and -resistant (W2) strains of *P. falciparum* (IC_50_ = 40 and 44 ng/mL respectively) with low cytotoxicity (6800 ng/mL) to mammalian kidney fibroblasts (Vero cells).

**Figure 4:**
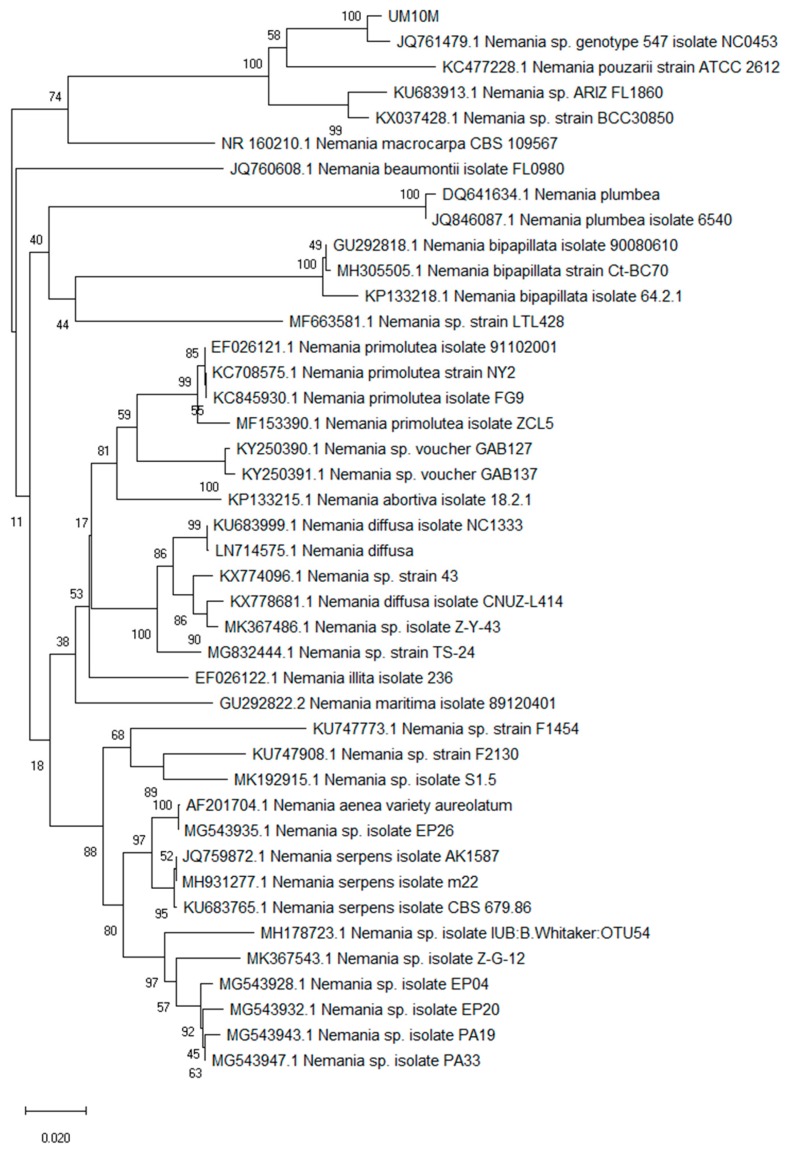
Constructed tree using Neighbor-Joining method employing MEGA X software to match UM10M to already published sequences to help identifying close relatives. Numbers displayed on branches are the percentage of replicate trees in which the associated taxa clustered together in the bootstrap test obtained through 500 replications. Sequences used are shown with GenBank accession numbers (Supplementary Table S1). UM10M is closely related to *Nemania* sp. genotype 547 isolate NC0453.

Analysis of the ITS genomic region (partial 18S ribosomal RNA gene, internal transcribed spacer 1, 5.8S ribosomal RNA gene, and internal transcribed spacer 2, and partial 28S ribosomal RNA gene) of this fungus (Figure 4 and Supplementary, Table S1) indicated that it is related to the genus *Nemania* of the family Xylariaceae with 545 of 557 nucleotides (98% sequence identity) to the *Nemania* sp. genotype 547 isolate NC0453 (GenBank accession: JQ761479.1) which represents an endolichenic fungus of the host *Pseudevernia consocians*. Since it does not show a 100% identity to other already published *Nemania* species in GenBank, this fungus was designated as a new isolate, UM10M (GenBank accession: MK321315).

The EtOAc extract of the culture broth of *Nemania* sp. UM10M was fractionated over silica gel and subsequently separated over Sephadex LH-20 and PTLC to afford three compounds. Compound **1** was identified as 19,20-epoxycytochalasin C by spectroscopic methods. This compound has previously been reported from several *Xylaria* species [24,25,26] and an unidentified endophytic fungus [27]. Our ^1^H and ^13^C NMR assignments were comparable to reported data, but the chemical shift for H-11 needs to be revised, and the ^13^C assignments for C-11 and C-12, and C-13 and C-14 need to be interchanged [25] (Table 1). Compound **2** has been isolated from *Xylaria* [25,28] *Engleromyces* [29,30], and an unidentified endophytic fungus [27], and its NMR data were comparable to the reported data [30].

Compound **3** (18-deoxy-19,20-epoxycytochalasin C) has been reported from an unidentified endophytic fungus, KL-1.1, and isolated from *Psidium guajava* leaves [27]. However, in the structure drawn for this compound, the absolute configurations of C-4 (*R*) and C-19 (*S*) were not correctly represented [30]. The NMR data for compound **3** were comparable to the reported data but the ^13^C assignments for C-12 and C-23 need to be interchanged (Table 1).

The configuration depicted in the reported structures for these compounds was not consistent [25,26,27,28,29,30]. In some cases, proper guidelines for using configurational descriptors have not been followed when drawing and defining stereogenic centers, and in others the absolute configuration of some stereogenic centers have been drawn incorrectly. The correct structure and the absolute configuration of 19,20-epoxycytochalasin D (**2**) have previously been determined by spectroscopic and X-ray analysis [30]. Our spectroscopic and physical data of compound **2** matched well with those reported for 19,20-epoxycytochalasin D [30], and we deduced that the structure in this reference represented the correct absolute configuration of compound **2**. Since compounds **1**–**3** presumably share the same biosynthetic precursor, they all possess the same absolute configuration. Therefore the structures and absolute configurations of these compounds were determined as (3*S*,4*R*,7*S*,8*S*,9*R*,16*S*,18*R*,19*R*,20*S*,21*S*)-19,20-epoxycytochalasin C (**1**), (3*S*,4*R*,5*S*,7*S*,8*S*,9*R*,16*S*,18*R*,19*R*,20*S*,21*S*)-19,20-epoxycytochalasin D (**2**), and (3*S*,4*R*,7*S*,8*S*,9*R*,16(*S*),18*S*,19*S*,20*R*,21*S*)-18-deoxy-19,20-epoxycytochalasin C (**3**) and are correctly represented in the structures shown in Figure 3.

Compounds **1**–**3** showed potent selective (calculated as a ratio of IC_50_ for cytotoxicity to Vero cells and IC_50_ for antimalarial activity) in vitro antiplasmodial activity against chloroquine-sensitive (D6) and -resistant (W2) strains of *P. falciparum* (Table 2) and nonspecific moderate phytotoxic activity against both a monocot (bentgrass, *Agrostis stolonifera*) and a dicot (lettuce, *Lactuca sativa cv.* L., iceberg) in the presence of light (Table 3).

The in vitro cytotoxic potential of **1**–**3** was further evaluated against a panel of solid tumor cell lines (SK-MEL, KB, BT-549, SK-OV-3) and kidney epithelial cells (LLC-PK_11_) (Table 4). Compounds **1** and **3** showed moderate toxicity to cell lines SK-MEL and BT-549, respectively, whereas compound **2** exhibited moderate toxicity to BT-549 and LLC-PK_11_ cell lines.

In vitro antiplasmodial [16,17] and antitoxoplasma [18] activities of cytochalasin derivatives have been reported. These compounds have been shown [17,18] to inhibit the actin-based gliding motility in extracellular parasites and impair erythrocyte invasion of apicomplexan parasites of *Toxoplasma gondii* [18,31] and *P. falciparum* [32,33]. Both host cells and the parasites contain actin-based cytoskeletons but due to their divergence, parasite actin-1 (PfACT-1) has been identified as a viable drug target [33]. The information on the activity of this class of compound indicates that the observed potent in vitro antimalarial activity with high selectivity indices for compounds **1** and **2** is most probably due to their selective disruption of parasite actin-1 and not due to disruption of plant-like metabolic pathways in apicoplasts. Even though in vitro antimalarial activity of this class of compounds has previously been reported [16,17], their in vivo activity has not yet been reported. Cytochalasins B, D, and E have been extensively studied for their anticancer effects. In animal models, no toxicity has been observed at the therapeutic doses when administered in intraperitoneal, subcutaneous, or intravenous injections up to 100 mg/kg/day [34,35,36].

Compound **1,** which was isolated as the major compound, was evaluated for antimalarial activity against *P. berghei* in mice at 100 mg/kg/day for 3 days through the oral route. Chloroquine was used as the positive control. A control group of mice were treated with vehicle only. Three out of 5 animals treated with compound **1** (Table 5) died due to drug induced toxicity. In animals that survived, 33.9% and 71.4% suppression of parasitemia was observed on days 5 and 7, respectively (Table 5). Even though compound **1** appears to have some suppressive activity at this dose, its high toxicity would preclude it from being a viable antimalarial lead.

## 3. Materials and Methods

### 3.1. General Experimental Procedures

Optical rotations were measured using a Autopol IV automatic polarimeter model 589-546 (Rudolph Research Analytical, Flanders, NJ, USA). NMR spectra were recorded on a Bruker 400 MHz. spectrometer (Rheinstetten, Germany) using CDCl_3_/methanol-*d*_4_ as the solvent unless otherwise stated. MS analyses were performed on an Agilent Series 1100 SL equipped with an ESI source (Agilent Technologies, Palo Alto, CA, USA). Column chromatography was carried out on silica gel 60 (230-400 mesh) (Sigma-Aldrich, St. Louis, MO, USA) and Sephadex LH-20 (GE Healthcare Bio-Sciences, Marlborough, MA, USA). Preparative TLC was carried out using silica gel GF plates (20 × 20 cm, thickness 0.25 mm). Spots were detected under UV light and by heating after spraying with anisaldehyde reagent.

### 3.2. Fungal Material

Diseased leaves were collected from a cultivated *T. taxifolia* plant from Oxford, MS. A voucher of the *T. taxifolia* Arn. plant was identified by E. M. Croom, Jr. and deposited in the University of Mississippi Pullen Herbarium. The voucher accession number is MISS 83490. Leaves were surface sterilized with 5% Chlorox for 5 min and rinsed with sterile water (×3). Transverse sections from the dried leaf were cut aseptically into small portions and immersed in potato dextrose plates. The plates were incubated for two weeks and fungal colonies were subcultured on PDA plates to isolate pure fungal strains.

### 3.3. Fermentation, Extraction, and Purification

Fungus UM10M was cultured in four conical flasks (2 L) containing 500 mL of PDB and incubated at 27 °C for 30 days on an orbital shaker at 100 rpm. Mycelia and broth were separated by filtration and extracted with EtOAc (×3). The organic layer from the broth was evaporated to give a brown/black residue (625 mg).

This extract (600 mg) was chromatographed on Sephadex LH-20 and eluted with 80% MeOH in CHCl_3_ to give 10 fractions. Fractions 2, 3, and 4 showed antimalarial activity. Fraction 2 was subjected to silica gel gravity column chromatography using hexanes, CH_2_Cl_2_, and MeOH gradient as the mobile phase to yield five fractions. Subfraction 3 which showed antimalarial activity was further separated by preparative thin layer chromatography (PTLC) using 0.4% MeOH in CHCl_3_ (×3) as the developing solvent to obtain 19,20-epoxycytochalasin C (**1**, 85.0 mg). Fractions 3 and 4 were also subjected to PTLC using 0.4% MeOH in CHCl_3_ (×3) as the developing solvent to afford 19,20-epoxycytochalasin D (**2**, 4 mg), and 18-deoxy-19,20-epoxycytochalasin C (**3**, 2 mg).

*19,20-Epoxycytochalasin C* (**1**); white amorphous powder; ^1^H, ^13^C NMR data (Table 1) ${\left[\alpha \right]}_{D}^{26}$ −13 (*c* 0.5, CHCl_3_) lit [24] −6.8; HRESIMS [M + H]^+^ *m*/*z* 524.2642.

*19,20-Epoxycytochalasin D* (**2**); white amorphous powder; ^1^H, and ^13^C NMR, data were consistent with literature values [30]. ${\left[\alpha \right]}_{D}^{26}$ −113 (*c* 0.1, CHCl_3_) lit [34] −190; HRESIMS [M + H]^+^ *m*/*z* 524.2837.

*18-Deoxy-19,20-epoxycytochalasin C* (**3**); white amorphous powder; ^1^H and ^13^C NMR (see Table 1) ${\left[\alpha \right]}_{D}^{26}$ −2.4 (*c* 0.1, CHCl_3_); HRESIMS [M + H]^+^ *m*/*z* 508.2703 (calcd for [C_24_H_33_NO_4_ + H]^+^ 508.26201.

### 3.4. Biological Assays

#### 3.4.1. In Vitro Antiplasmodial Assay

The antiplasmodial assay was performed against D6 (chloroquine sensitive) and W2 (chloroquine resistant) strains of *P. falciparum* using the in vitro assay as reported [37]. Artemisinin and chloroquine were included as the drug controls, and IC_50_ values were computed from the dose-response curves.

#### 3.4.2. In Vitro Phytotoxicity Assay

Herbicidal or phytotoxic activity of the extract and compounds was performed according to the published procedure [38] using bentgrass (*Agrostis stolonifera*) and lettuce (*Lactuca sativa* cv. L., Iceberg), in 24-well plates. Phytotoxicity was ranked visually. The ranking of phytotoxic activity was based on a scale of 0 to 5 with 0 showing no effect and 5 no growth.

#### 3.4.3. In Vitro Cytotoxicity Assay

In vitro cytotoxicity was determined against a panel of mammalian cells that included kidney fibroblast (Vero), kidney epithelial (LLC-PK_11_), malignant melanoma (SK-MEL), oral epidermal carcinoma (KB), breast ductal carcinoma (BT-549), and ovary carcinoma (SK-OV-3) cells [39] Doxorubicin was used as a positive control.

#### 3.4.4. In Vivo Antimalarial Assay

The protocol for in vivo antimalarial evaluation was approved by the University of Mississippi Institutional Animal Care and Use Committee (IACUC). The in vivo antimalarial activity was determined in mice infected with *P. berghei* (NK-65 strain). Male mice (Swiss Webster strain) weighing 18-20 g were intraperitoneally inoculated with 2 × 10^7^ parasitized red blood cells obtained from a highly infected donor mouse. Mice were divided into different groups with five mice in each group. Compound stocks were prepared in DMSO and administered orally to the mice through gavage two hours after the infection (day 0). The animals were treated once daily for consecutive days (day 0 to 2) and were closely observed for at least 2 h after every dose for any apparent signs of toxicity. Blood smears were prepared on different days starting from 5 days post infection (through 28 days) by clipping the tail end, stained with Giemsa, and the slides were observed under microscope for determination of parasitemia. Mice without parasitemia through day 28 postinfection were considered as cured. Also, suppression in development of parasitemia was computed by comparing the parasitemia in the control vehicle treated group and groups treated with compound. The mean survival time was also computed for control and treated groups. The results are presented as parasitemia suppression of day 5 post treatment, mean survival time of mice in each group, cure and survival graphs computed by Prism 6.0. (San Diego, CA, USA).

### 3.5. DNA Analysis

A 0.5 by 1.5 cm piece of fungus grown on solid media was transferred into a 2 mL microcentrifuge tube. Two 3 mm in diameter stainless steel balls were added and the sample ground for 1 min in a MM2000 mixer mill (Retsch, Haan, Germany). DNA was extracted with the DNeasy Plant mini kit (Qiagen, Valencia, CA, USA) according to the manufacturer’s recommendation. DNA was eluted with 50 μL buffer AE. DNA quality and quantity was determined with the NanoDrop 1000 Spectrophotometer (Thermo Fisher, Wilmington, DE, USA). DNA dilutions were prepared to achieve a 10 ng/μL solution to be used as template for PCR.

The internal transcribed spacer region (ITS) was amplified in a 25 μL reaction containing 10 ng DNA, 1X PCR reaction buffer, 0.2 mM dNTP mixture, 0.2 μM of each forward and reverse primers ITS1 and ITS4 [40], 1.5 mM MgSO_4_ and 1 U of Taq Polymerase High Fidelity (Invitrogen, Carlsbad, CA, USA). The PCR program consisted of 40 cycles with denaturation at 94 °C for 3 min, 50 °C annealing temperature for 30 s, 68 °C for 90 s, and a final extension at 68 °C for 3 min. After amplification, an aliquot was analyzed by electrophoresis on a 0.7% TAE agarose gel. Successfully amplified PCR products were cleaned up with MinElute PCR Purification Kit (Qiagen) according to the manufacturer’s instructions. PCR products were cloned into pJet.1.2 blunt using the CloneJet PCR cloning kit (Fermentas, Glen Burnie, MD, USA). Eight individual colonies were transferred into 4 mL liquid LB carbenicillin media and grown under constant shaking overnight at 37 °C. Plasmid DNA from the overnight cultures was isolated with the Qiagen plasmid purification kit (Qiagen) according to the manufacturer’s instructions. Sequencing was performed at the Genomics and Bioinformatics Research Facility in Stoneville, MS. Resulting sequences were analyzed with DNASTAR (DNASTAR, Madison, WI, USA). Homology searches were performed with the Basic Local Alignment Search Tool (BLAST) [41]. The UM10M sequence was submitted to GenBank (Accession: MK321315).

To visualize sequence alignment the software Genedoc [42] was used. Shading is according to alignment consensus as given by GeneDoc (black, 100%; dark grey, 80%; light grey, 60%) (Supplementary, Figure S19). To identify close relatives of UM10M a phylogenetic analysis was performed (Supplementary, Figure S19) using three best hits from the BLAST analysis and already published sequences in Genebank of various taxa of the genus *Nemania*. The configuration for the alignment analysis was maximum likelihood with the neighbor joining statistical method [43]. The percentage of replicate trees in which the associated taxa clustered together in the bootstrap test (500 replicates) is shown next to the branches [44]. The evolutionary distances were computed using the Kimura 2-parameter method [45] with nucleotide transitions and transversions included and are in the units of the number of base substitutions per site. The analysis involved 42 nucleotide sequences (Supplementary Table S1). All positions containing gaps and missing data were eliminated by the software for the final dataset analysis. There were a total of 692 positions in the final dataset. Evolutionary analyses were conducted in MEGA X [46].

## 4. Conclusions

Three cytochalasins were isolated from an endophytic fungus (UM10M) of the genus *Nemania* identified from a diseased leaf of *T. taxifolia* Arnott. All compounds showed potent in vitro activity against *P. falciparum* D6 and W2 strains with no cytotoxicity to Vero cells but displayed moderate to weak cytotoxicity to some of the human solid tumor cell lines and kidney epithelial cells. Compound **1** was evaluated in a mouse model for activity against *P. berghei* at 100 mg/kg/day. It showed weak suppressive activity but its high toxicity to animals would preclude it as a potential malaria drug lead.

## Figures and Tables

**Table 1:** ^1^H and ^13^C NMR data of **1** and **3** in CDCl_3_-methanol-*d*_4_.

Carbon	1		3	
	*δ*_C_^b^	*δ*_H_^a^ (*J* in Hz)	*δ*_C_^b^	*δ*_H_^a^ (*J* in Hz)
1	175.0		175.1	
3	61.0	3.35, t (7.3)	61.1	3.32, m
4	49.9	2.50, brs	49.9	2.43, brs
5	126.4		128.5	
6	131.8		131.7	
7	68.1	3.76, brd (9.9)	68.1	3.77, d (9.7)
8	48.7	2.25, dd (10.1,10.0)	49.0	2.27, dd, (10.1, 10.1)
9	52.0		51.8	
10	44.1	3.03, d (7.4)	44.4	3.04, bd, (7.5)
11	16.7	1.19, s	14.1	1.26, s
12	13.8	1.62, s	13.9	1.65, s
13	131.4	5.99, dd (15.5, 10.2)	131.0	6.24, dd (15.5, 10.4)
14	132.9	5.68, ddd (15.6, 9.7, 5.8)	133.7	5.61, m
15	37.5	2.63, dd (22.7, 11.9), 2.14, m ^c^	37.6	2.53, dd (25.3, 12.2), 2.14 m ^c^
16	41.7	3.27, m ^c^	42.9	2.98, d (1.8)
17	215.3		216.9	
18	76.3		52.1	2.18, m ^c^
19	59.9	3.25, brs ^c^	58.6	3.01, m ^c^
20	53.2	3.38, brs	57.6	3.38, d (1.7)
21	72.0	5.75, s	72.3	5.69, s
22	18.9	1.21, d (7.6)	18.8	1.12, d (6.7)
23	21.5	1.54, s	17.1	1.32, d (6.9)
24	170.4		170.5	
25	20.5	2.18, s	20.7	2.15, s
1′	137.3		136.9	
2′,6′	129.2	7.28, m	129.4	7.26, m
3′,5′	128.6	7.33, m	128.7	7.33, m
4′	126.8	7.25, m	126.9	7.25, m

^a 1^H NMR spectra recorded at 400 MHz, ^b 13^C NMR spectra recorded at 100 MHz, ^c^ overlapped signal.

**Table 2:** Antiplasmodial activity of **1**–**3**.

Compound	Chloroquine-Sensitive (D6)-Strain		Chloroquine-Resistant (W2)-Strain		Cytotoxicity to Vero Cells
	IC_50_ µM (ng/mL)	S. I.	IC_50_ µM (ng/mL)	S. I.	IC_50_ µM
19,20-Epoxycytochalasin C (**1**)	0.07 (37)	>128.6	0.05 (28)	>170	NC
19,20-Epoxycytochalasin D (**2**)	0.04 (22)	>216.3	0.04 (20)	>238	NC
18-Deoxy-19,20-epoxy-cytochalasin C (**3**)	0.56 (280)	>17	0.19 (100)	>47.6	NC
Chloroquine ^a^	0.03 (16)	>297.5	0.31 (160)	>29.8	NC
Artemisinin ^a^	0.02 (5.6)	>850	0.01 (3.0)	>1586.6	NC

^a^ Positive controls; NC: not cytotoxic at the highest concentration tested (4760 ng/mL); S. I. (selectivity index) = IC_50_ for cytotoxicity/IC_50_ for antiplasmodial activity.

**Table 3:** Phytotoxic activity of **1**–**3** ^a^.

Compound	Lettuce	Bentgrass
19,20-Epoxycytochalasin C (**1**)	3	2
19,20-Epoxycytochalasin D (**2**)	3	3
18-Deoxy-19,20-epoxycytochalasin C (**3**)	3	4

^a^ Concentration (mM) = 1 mg/mL. Ranking based on scale of 0 to 5; 0 = no effect; 5 = no growth;Solvent used, 10% acetone; Concentration used, 1 mg/mL.

**Table 4:** Cytotoxic activity [IC_50_ (µM)] of **1**–**3**.

Compound	SK-MEL	KB	BT-549	SK-OV-3	LLC-PK_11_
19,20-Epoxycytochalasin C (**1**)	8.02	NC	NC	NC	NC
19,20-Epoxycytochalasin D (**2**)	NC	NC	7.84	NC	8.4
18-Deoxy-19,20-epoxycytochalasin C (**3**)	NC	NC	6.89	NC	NC
Doxorubicin ^a^	1.29	2.12	1.83	1.47	1.28

^a^ Positive control. NC: not cytotoxic at 10 µM. IC_50_ = concentration causing 50% growth inhibition. SK-MEL = human malignant melanoma; KB = human epidermal carcinoma; BT-549 = human breast carcinoma (ductal); SK-OV-3 = human ovary carcinoma; LLC-PK_11_ = pig kidney epithelial.

**Table 5:** In vivo antimalarial activity of **1**.

Treatment (PO)	Dose (mg/kg × # days Post Infection)	% Parasitemia Suppression ^b^		Survival ^c^	Day of Death	MST ^d^	Cure ^f^
		Day 5	Day 7				
Vehicle	×3	-	-	0/5	14/14/13/13/5	11.8	0/5
Chloroquine ^a^	100 × 3	100	100	5/5	28/28/28/28/28	28	2/5
**1**	100 × 3	33.9	71.4	0/5	17/5/3/1/0	5.2	0/2

^a^ Positive control; ^b^ % suppression in parasitemia is calculated by considering the mean parasitemia in the vehicle control as 100%, Parasitemia suppression < 80% is considered as non-significant; ^c^ Number of animals that survived day 28/total animals in group (the day of the death-post infection); ^d^ % MST—mean survival time (days); ^f^ Number of mice without parasitemia (cured) till day 28 post-infection.

## Supplementary Materials

The following are available online. Figures S1–S18 are NMR spectra of compounds **1**–**3**, Figure S19 is UM10M sequence alignment, Table S1 is Sequences used for alignment analysis and to identify close relatives.
