# Supplementary material for: Antiplasmodial and Cytotoxic Cytochalasins from an Endophytic Fungus, Nemania sp. UM10M, Isolated from a Diseased Torreya taxifolia Leaf
Source: Molecules. 2019 Feb 21;24(4):777. doi: 10.3390/molecules24040777 (PMC6413121; doi:10.3390/molecules24040777)
Supplement: Supplementary file 1 [file molecules-24-00777-s001.pdf]

Supplementary Information

Antiplasmodial and Cytotoxic Cytochalasins from an Endophytic Fungus, *Nemania* sp. UM10M, Isolated from a Diseased *Torreya taxifolia* Leaf

Mallika Kumarihamy[1,2], Daneel Ferreira[2], Edward M. Croom Jr.[2], Rajnish Sahu[1], Babu L. Tekwani[1], Stephen O. Duke[3], Shabana Khan[1,2], Natascha Techen[1], and N. P. Dhammika Nanayakkara[1,*]

[1] National Center for Natural Products Research, Research Institute of Pharmaceutical Sciences, University of Mississippi, Mississippi, 38677, USA; mkumarih@olemiss.edu (M.K.); rsahu@alasu.edu (R.S); btekwani@southenresearch.org (B.L.T); skhan@olemiss.edu (S.K.); ntechen@olemiss.edu (N.T.); dhammika@olemiss.edu (D.N.);

[2] Department of BioMolecular Sciences, Division of Pharmacognosy, School of Pharmacy, The University of Mississippi, University, MS 38677, USA; dferreir@olemiss.edu (D.F); emcroom@olemiss.edu (E.M.C.)

[3] Natural Products Utilization Research Unit, USDA-ARS, University, MS 38677, USA; stephen.duke@ars.usda.gov

***** Correspondence: dhammika@olemiss.edu; Tel.: +1-662-915-1019

**Contents**	**Figure**	**Table**
^{1}H NMR Spectrum of compound **1**	S1	
^{13}C NMR Spectrum of compound **1**	S2	
DEPT Spectrum of compound **1**	S3	

COSY Spectrum of compound **1** S4

HMQC Spectrum of compound **1** S5

HMBC Spectrum of compound **1** S6

^{1}H NMR Spectrum of compound **2** S7

^{13}C NMR Spectrum of compound **2** S8

DEPT Spectrum of compound **2** S9

COSY Spectrum of compound **2** S10

HMQC Spectrum of compound **2** S11

HMBC Spectrum of compound **2** S12

^{1}H NMR Spectrum of compound **3** S13

^{13}C NMR Spectrum of compound **3** S14

DEPT Spectrum of compound **3** S15

COSY Spectrum of compound **3** S16

HMQC Spectrum of compound **3** S17

HMBC Spectrum of compound **3** S18

UM10M Sequence Alignment ` S19

Sequences used for alignment analysis and to identify close relatives S1

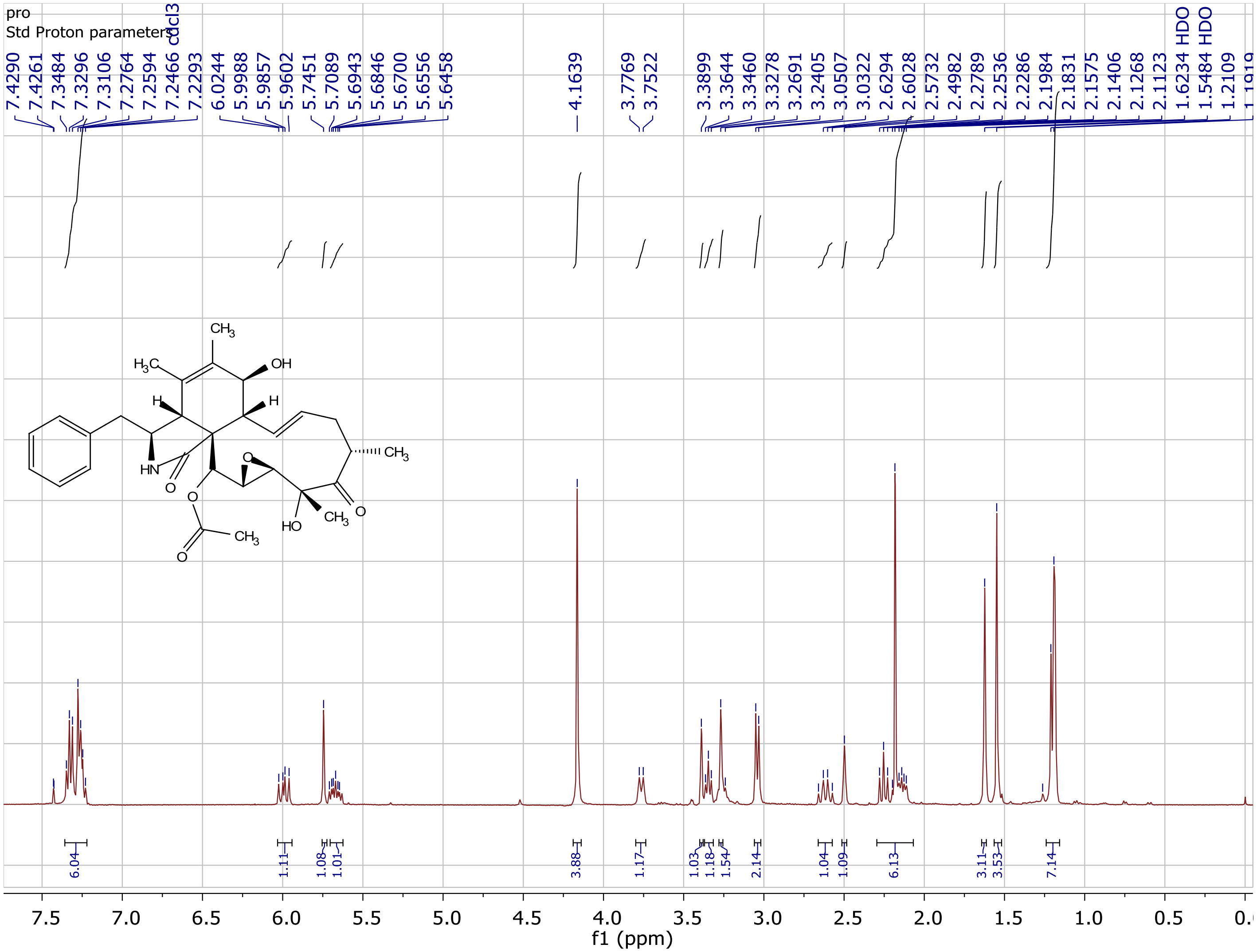

Figure S1: ^{1}H NMR Spectrum of compound **1**

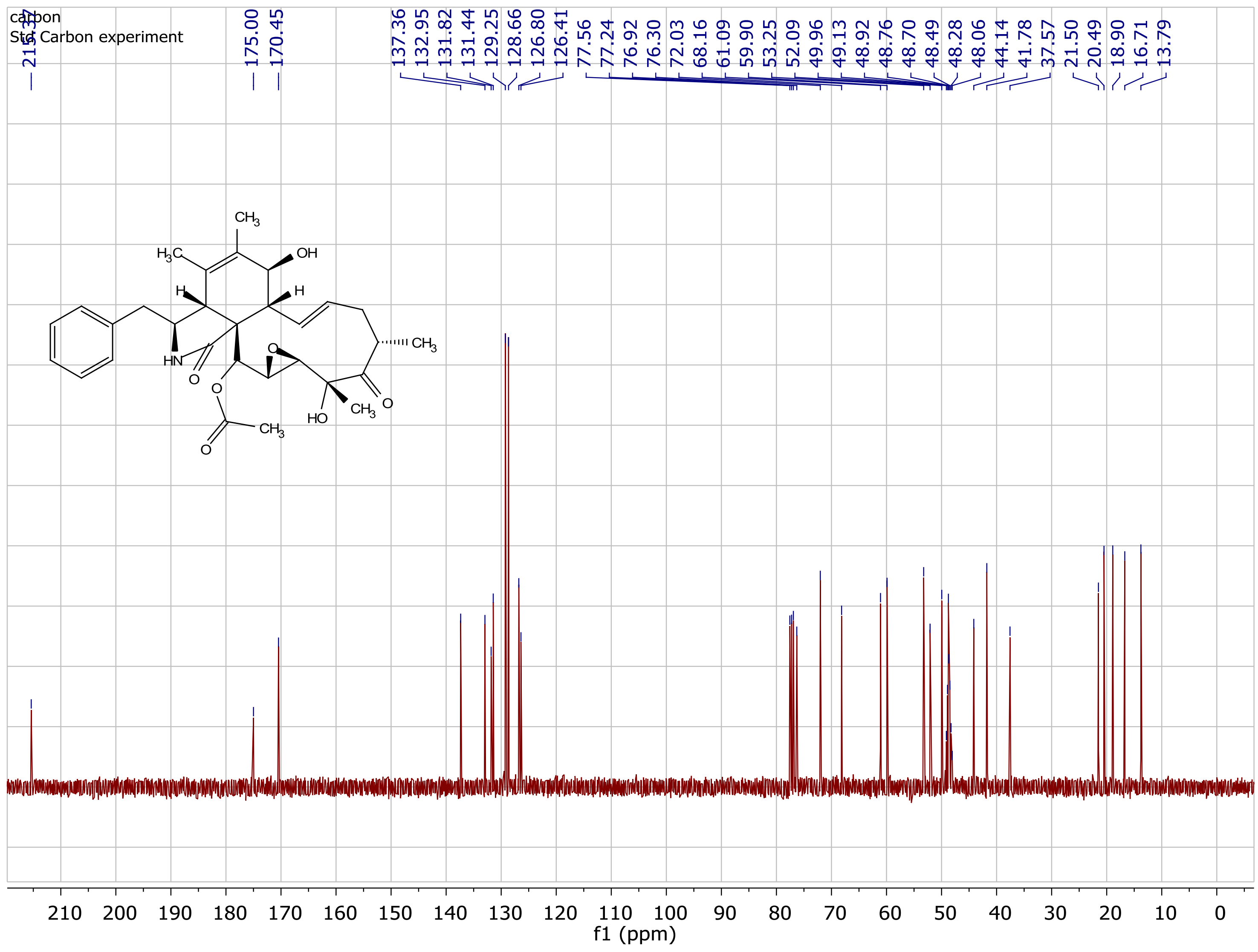

Figure S2: ^{13}C NMR Spectrum of compound **1**

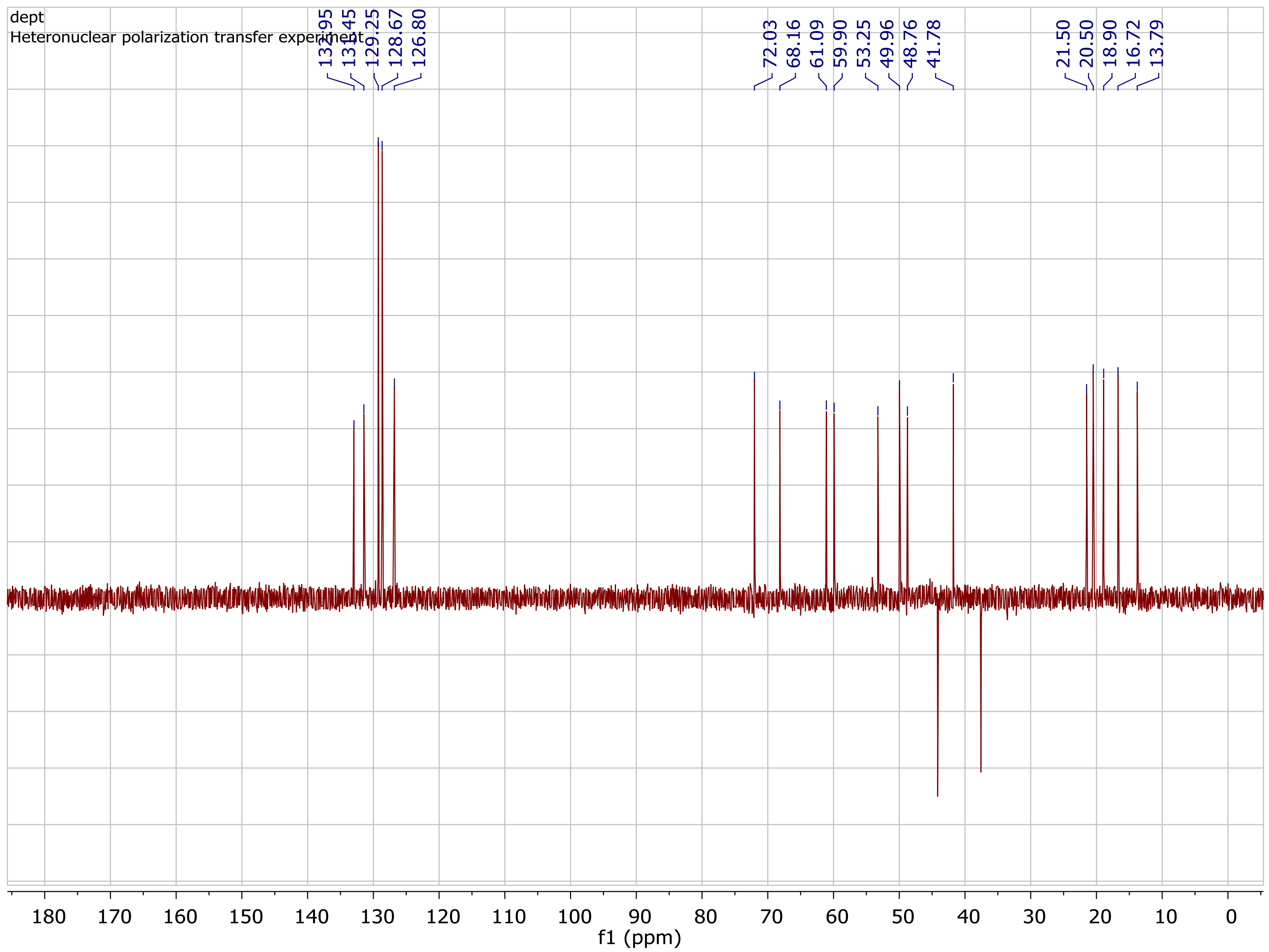

Figure S3: DEPT Spectrum of compound **1**

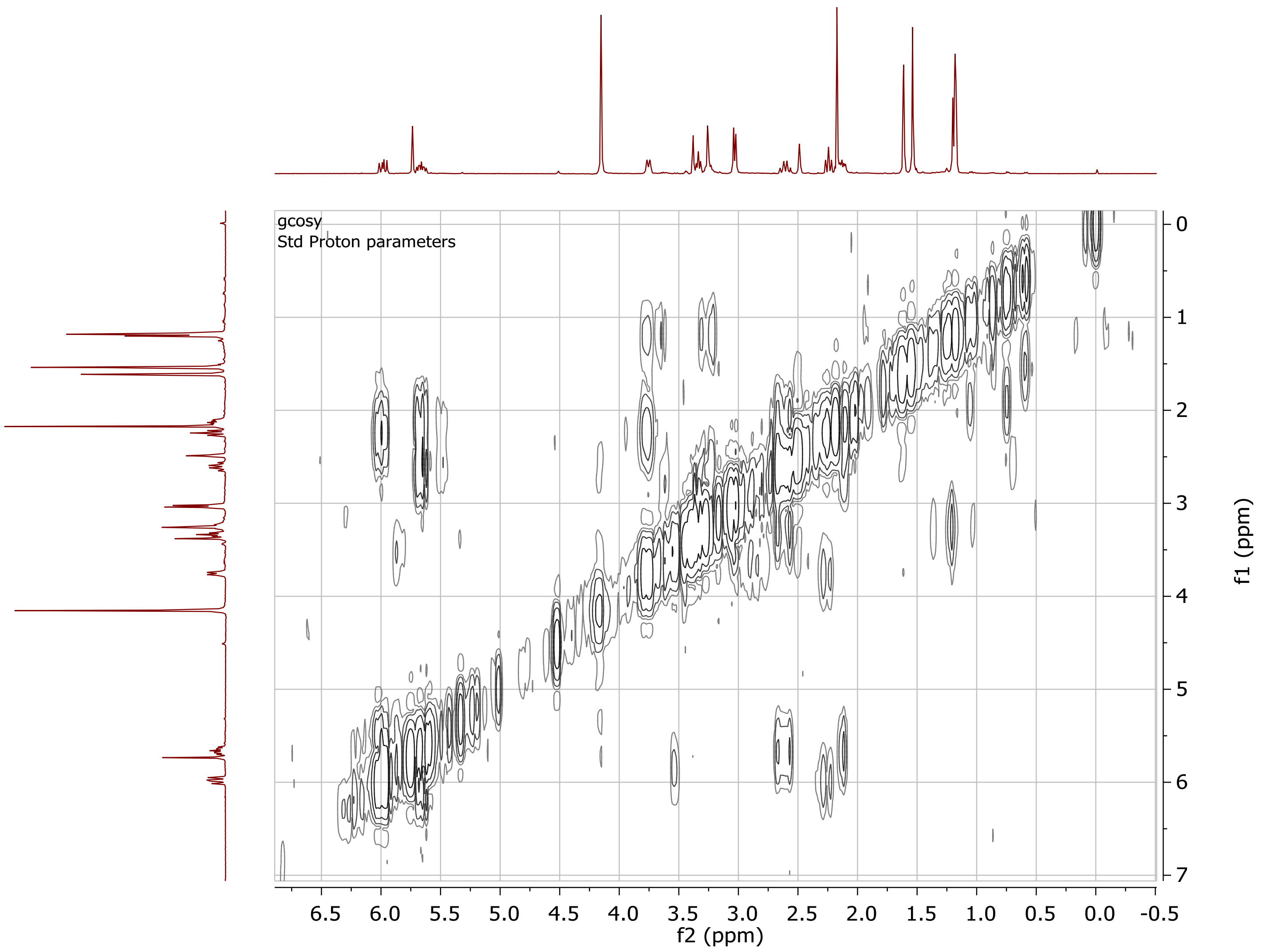

Figure S4: COSY Spectrum of compound **1**

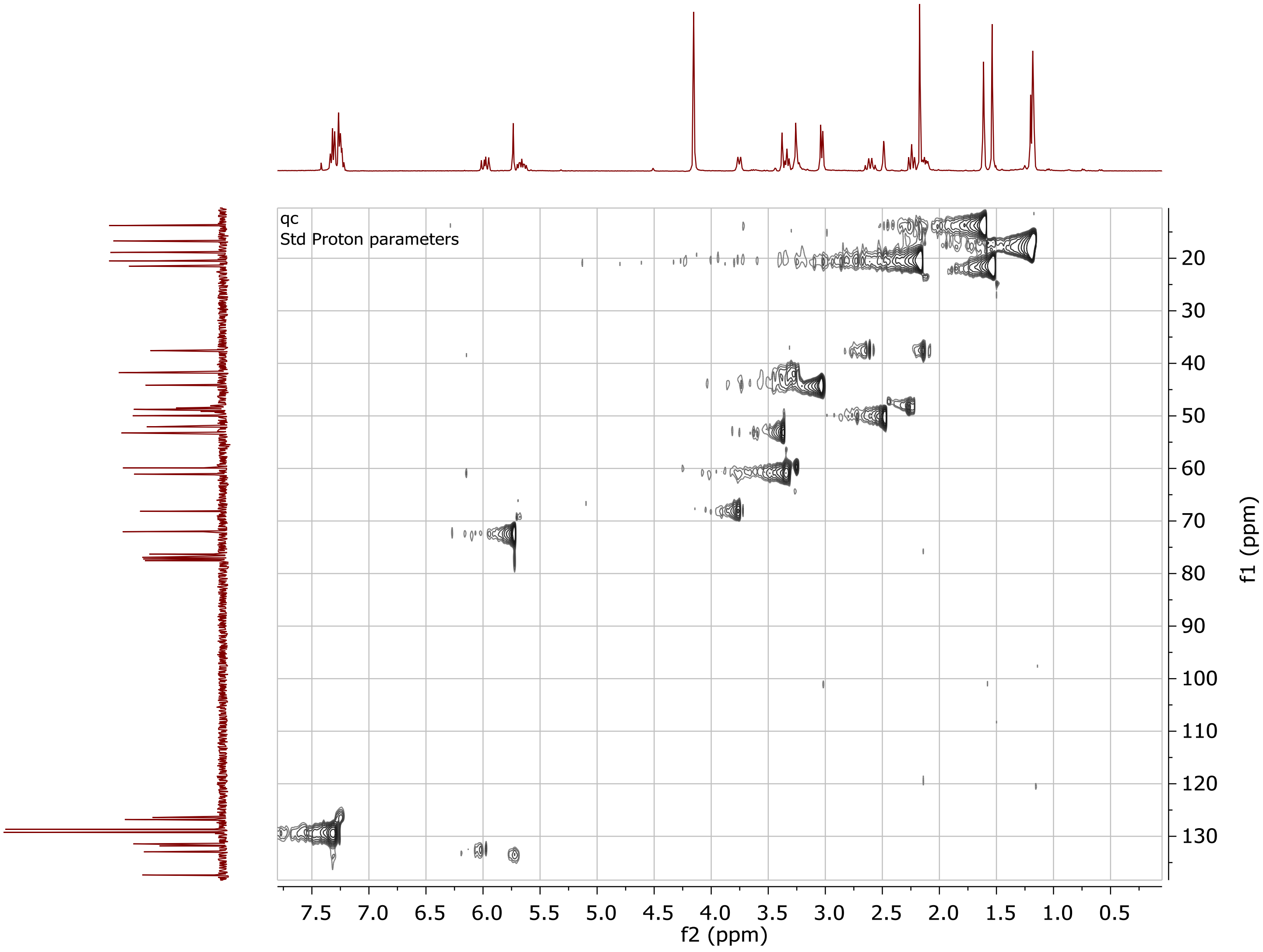

Figure S5: HMQC Spectrum of compound **1**

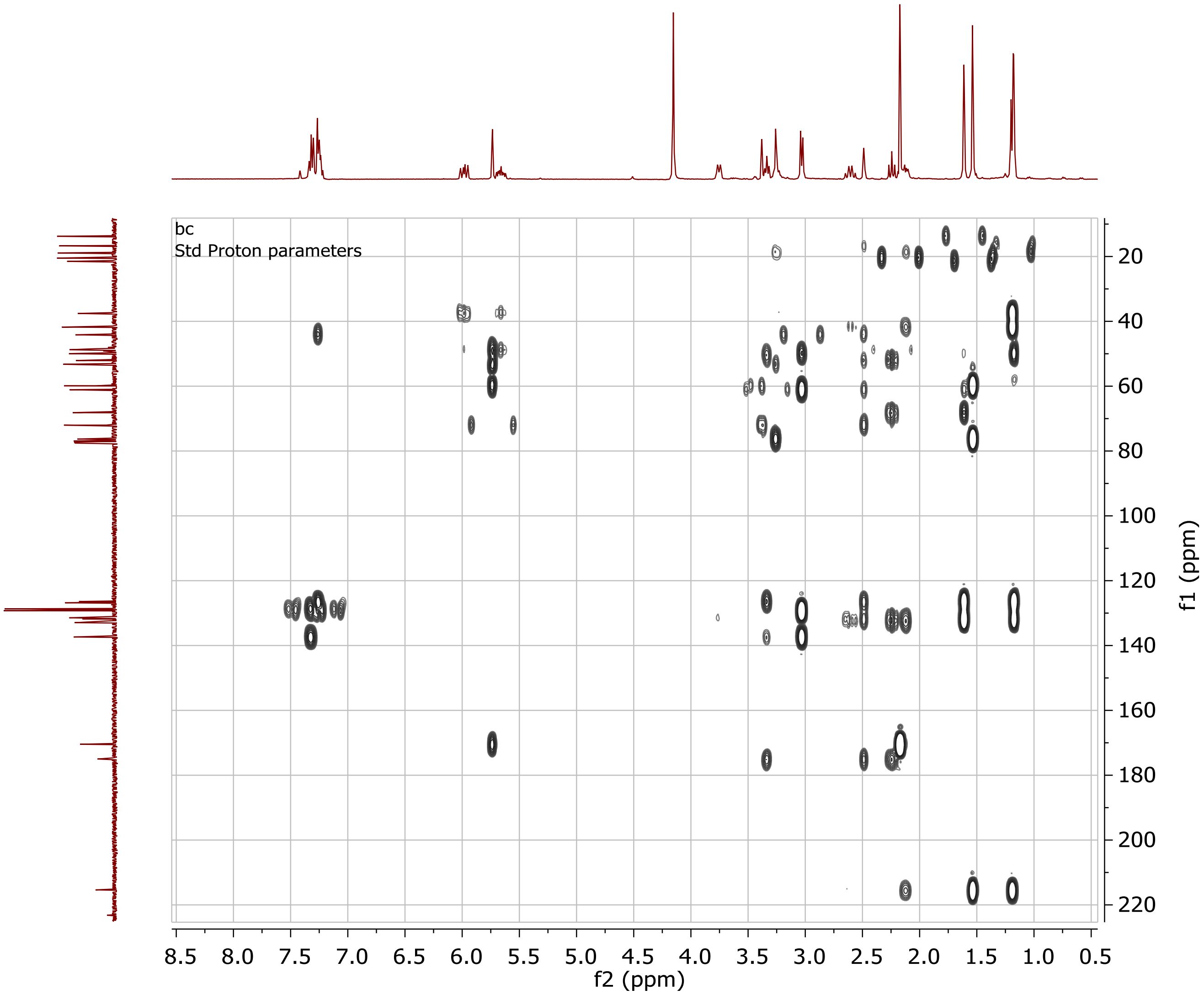

Figure S6: HMBC Spectrum of compound **1**

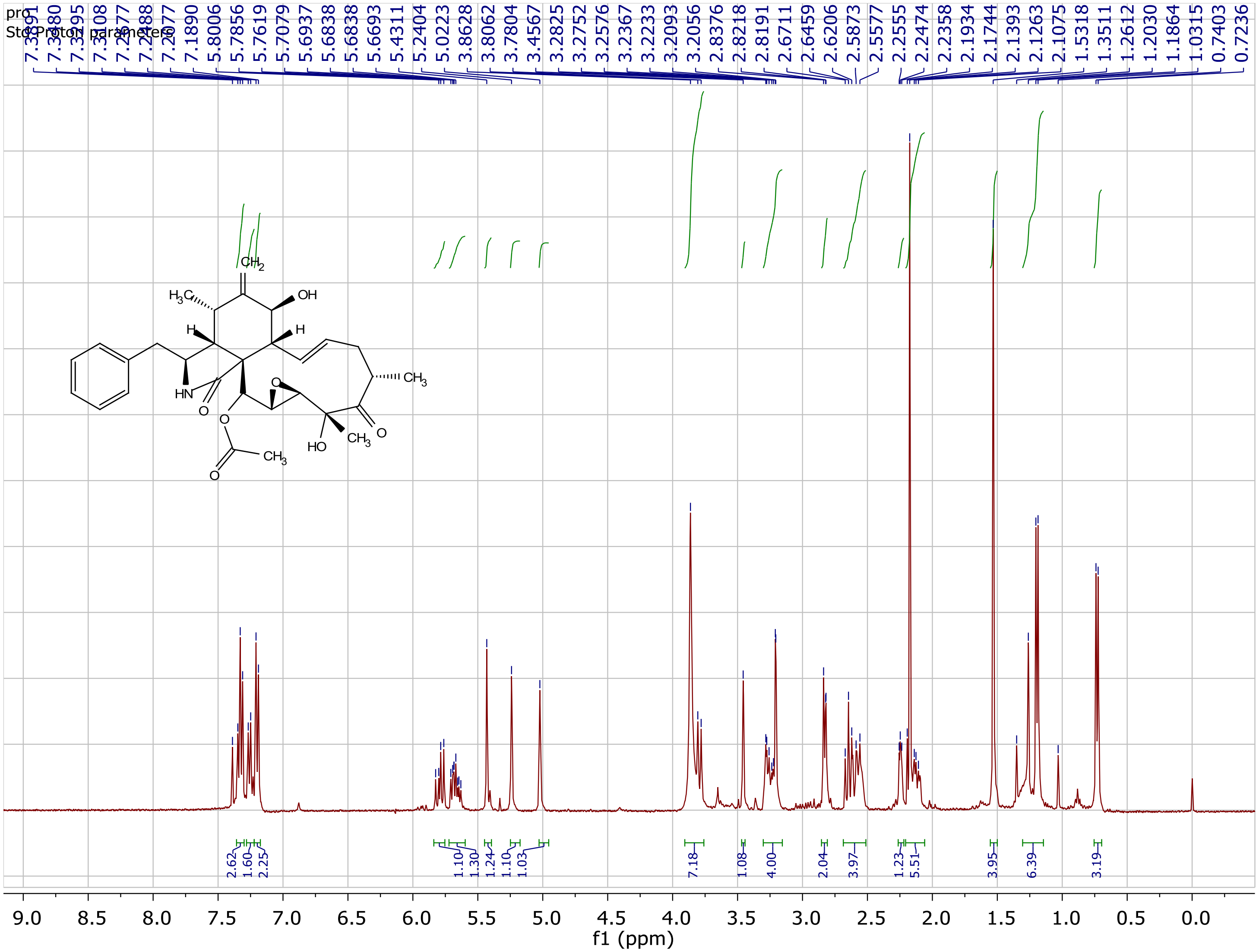

Figure S7: ^{1}H NMR Spectrum of compound **2**

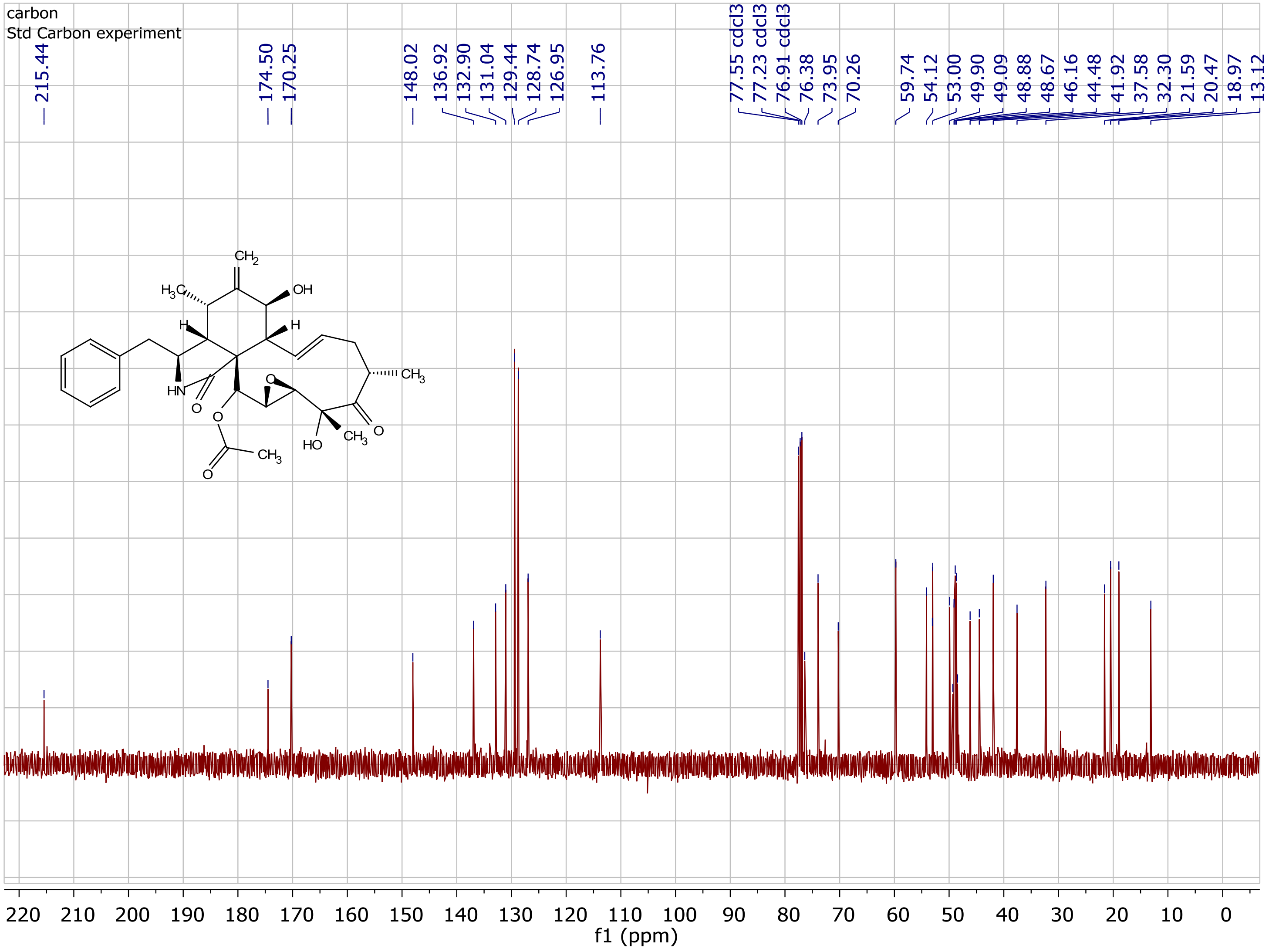

Figure S8: ^{13}C NMR Spectrum of compound **2**

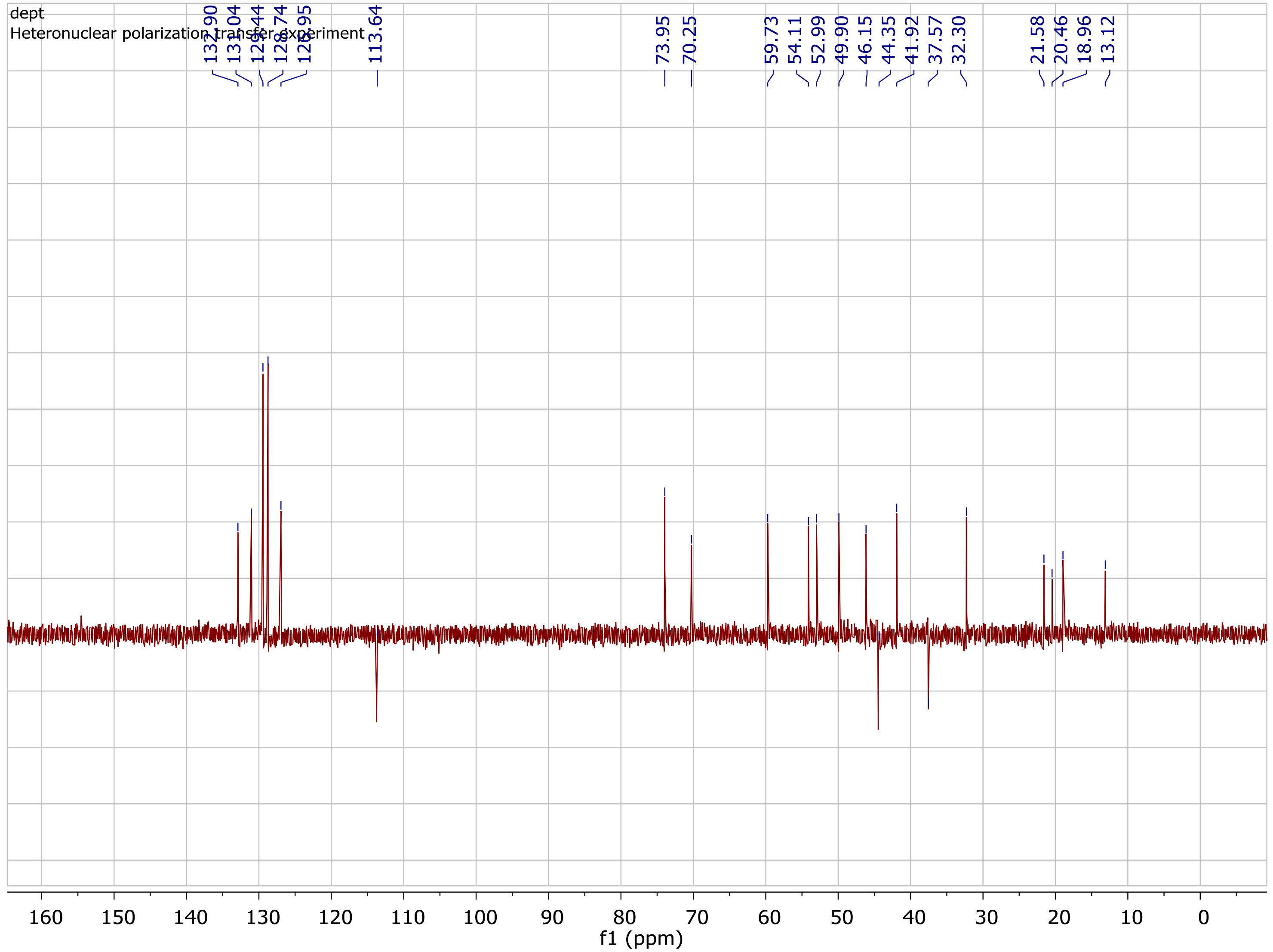
dept
Heteronuclear polarization transfer experiment
132.90
131.04
129.44
128.74
126.95
113.64
73.95
70.25
59.73
54.11
52.99
49.90
46.15
44.35
41.92
37.57
32.30
21.58
20.46
18.96
13.12
160 150 140 130 120 110 100 90 80 70 60 50 40 30 20 10 0
f1 (ppm)

Figure S9: DEPT Spectrum of compound **2**

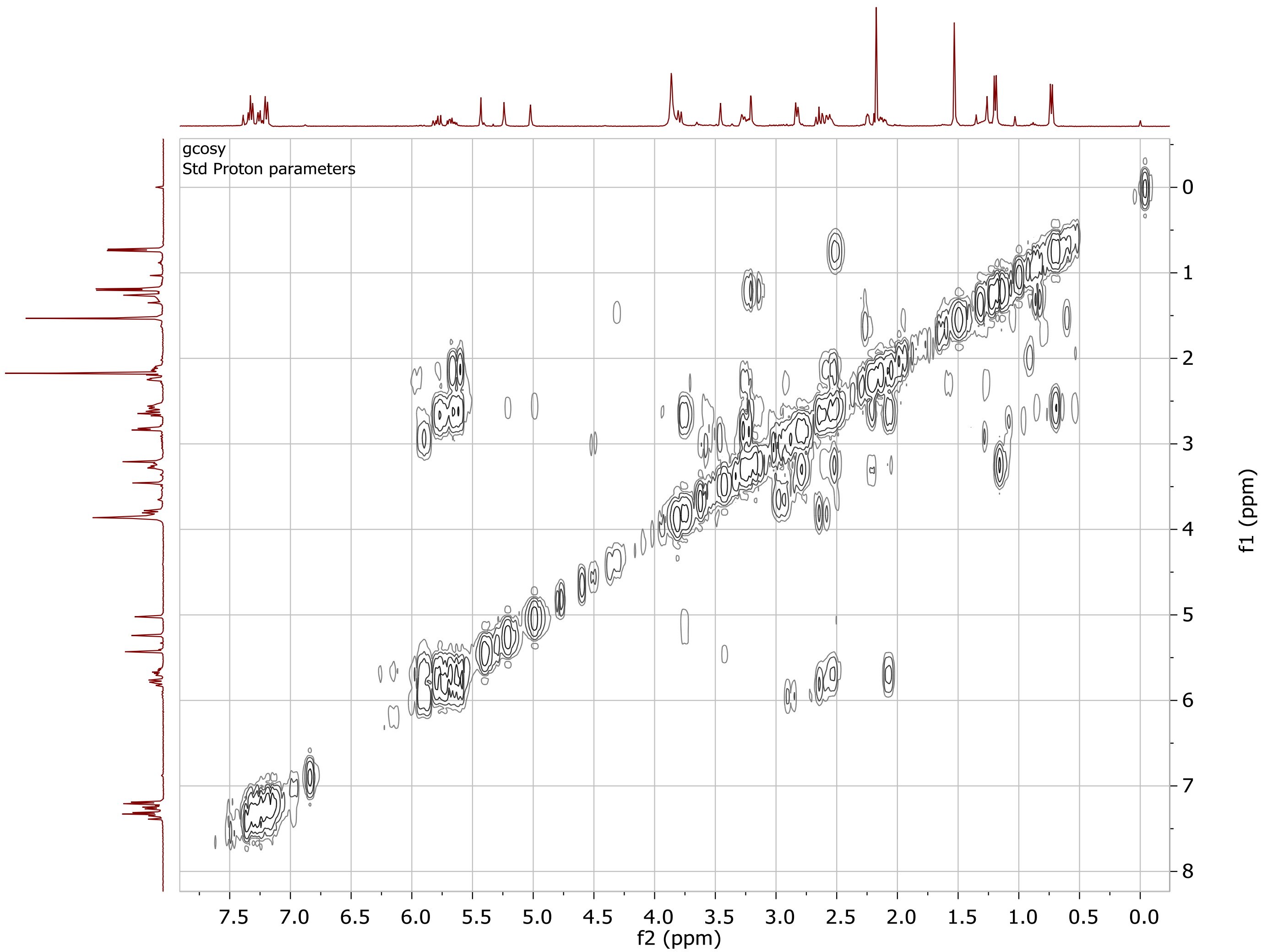

Figure S10: COSY Spectrum of compound **2**

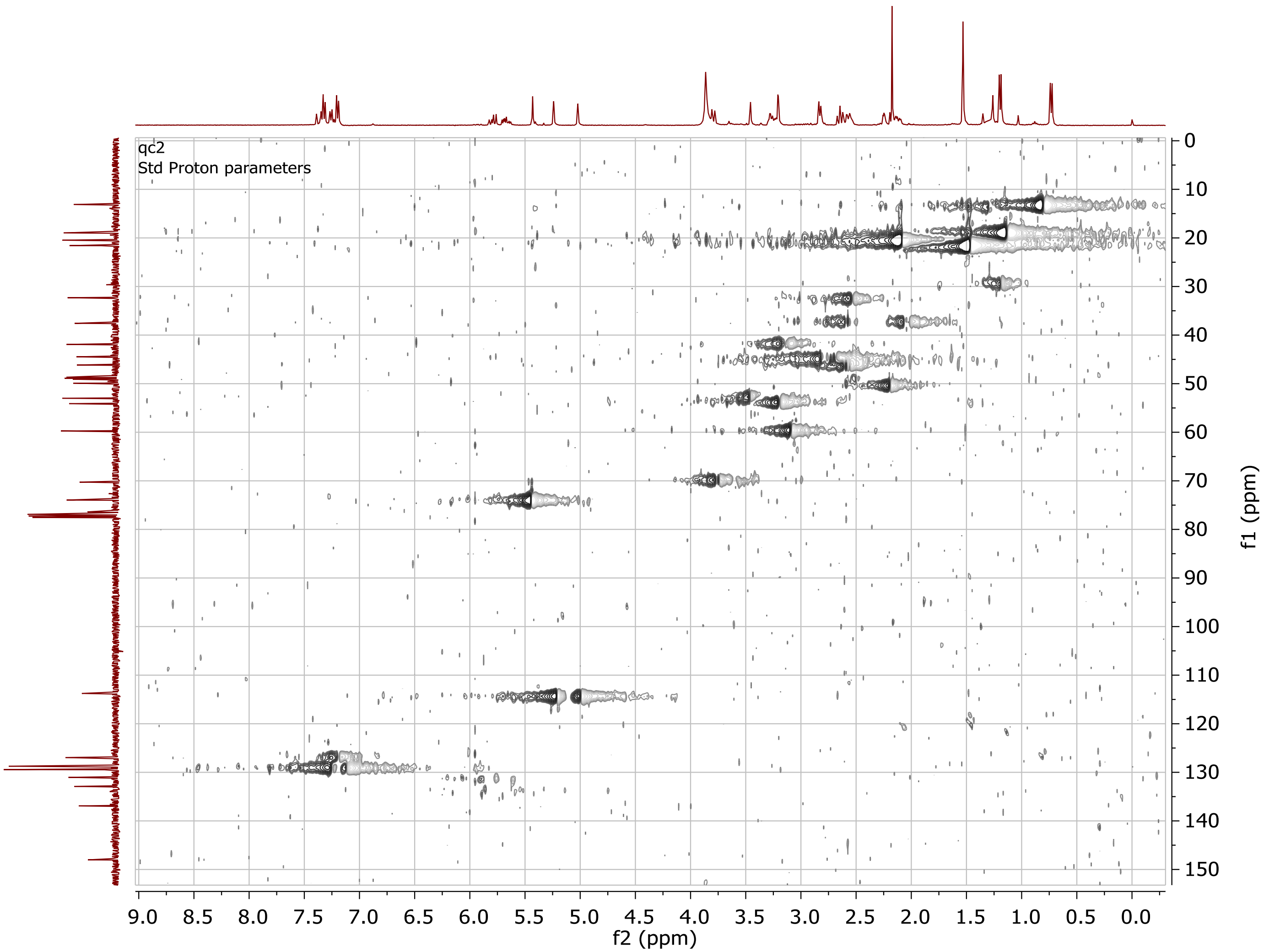

Figure S11: HMQC Spectrum of compound **2**

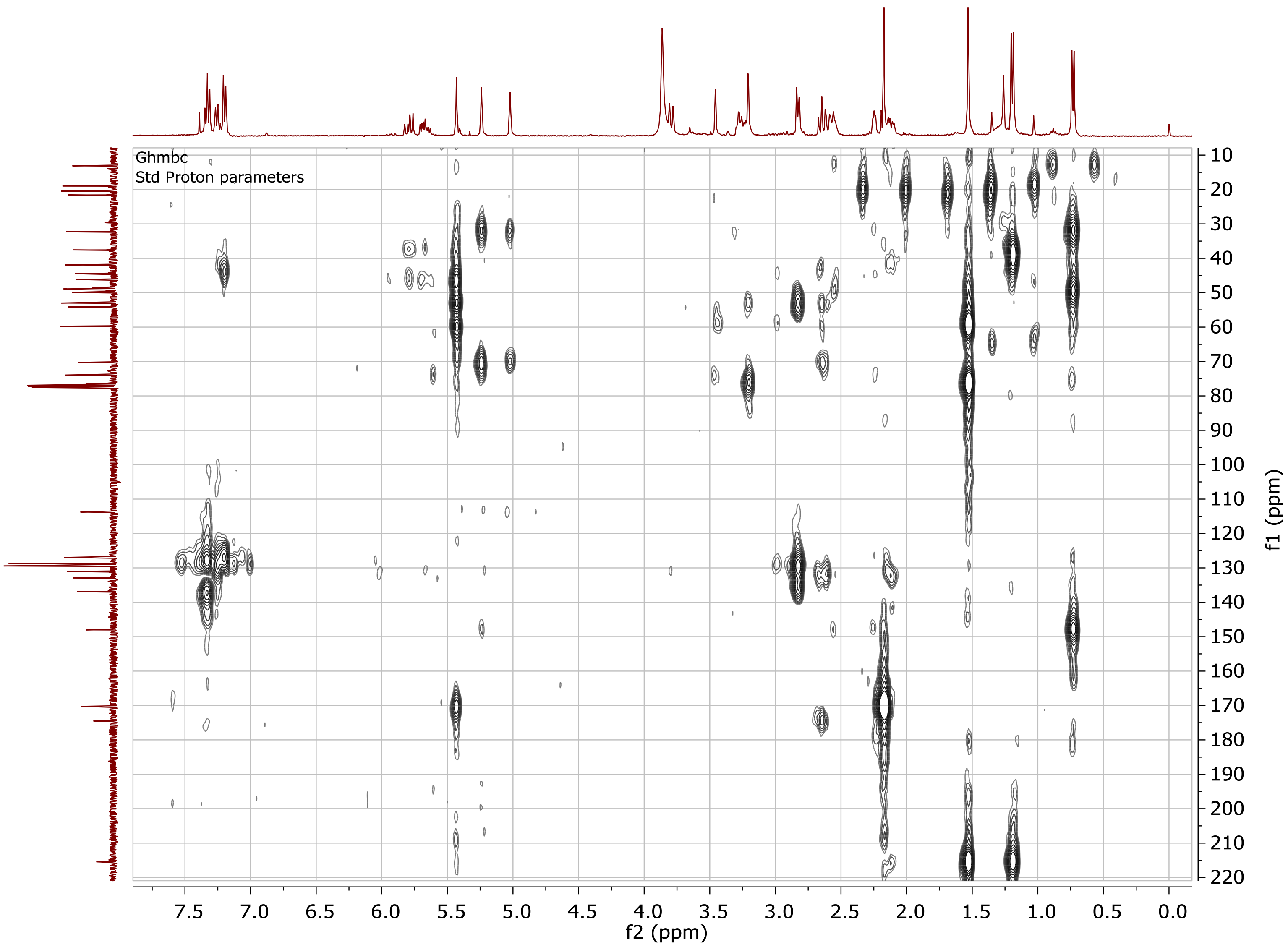

Figure S12: HMBC Spectrum of compound **2**

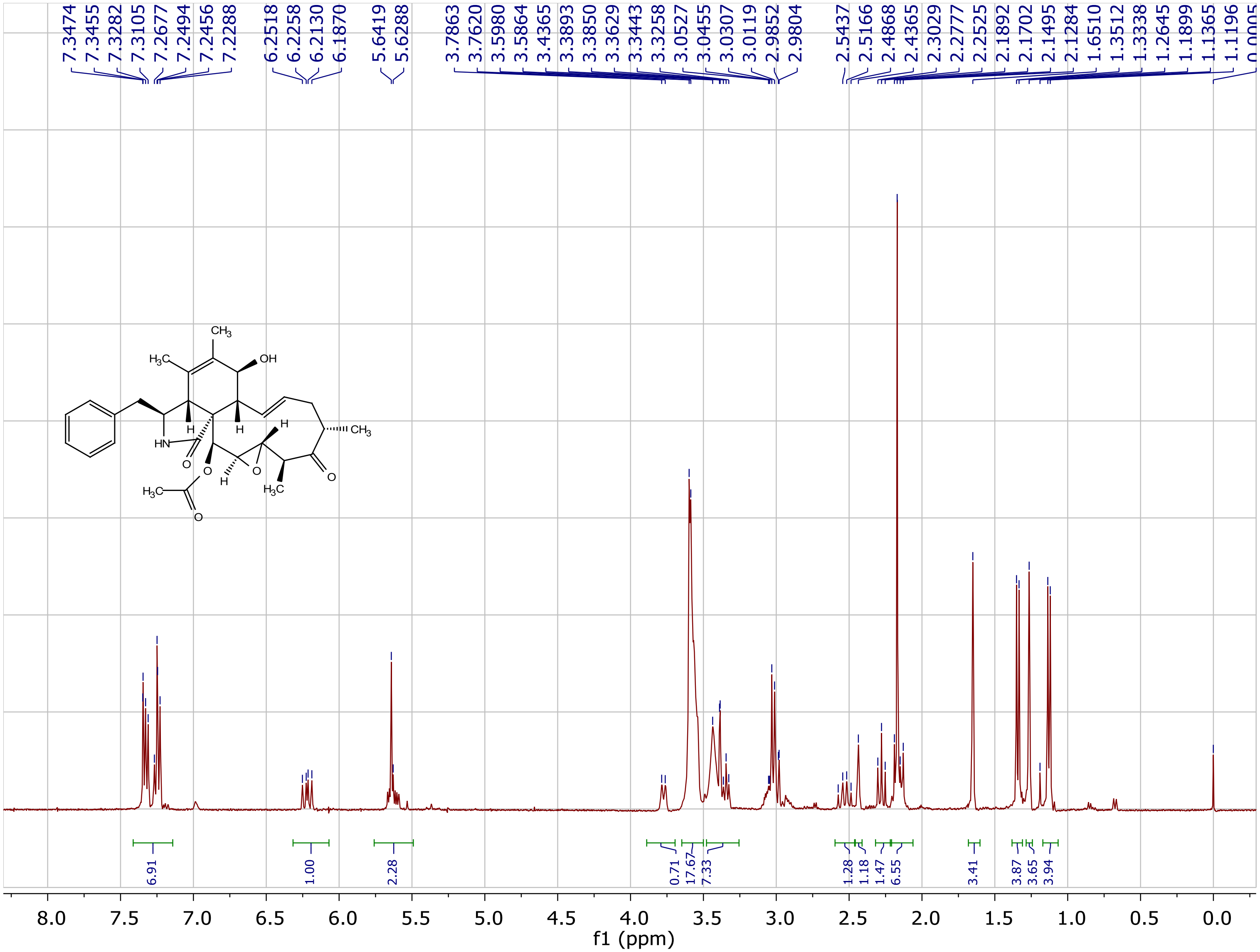

Figure S13: ^{1}H NMR Spectrum of compound **3**

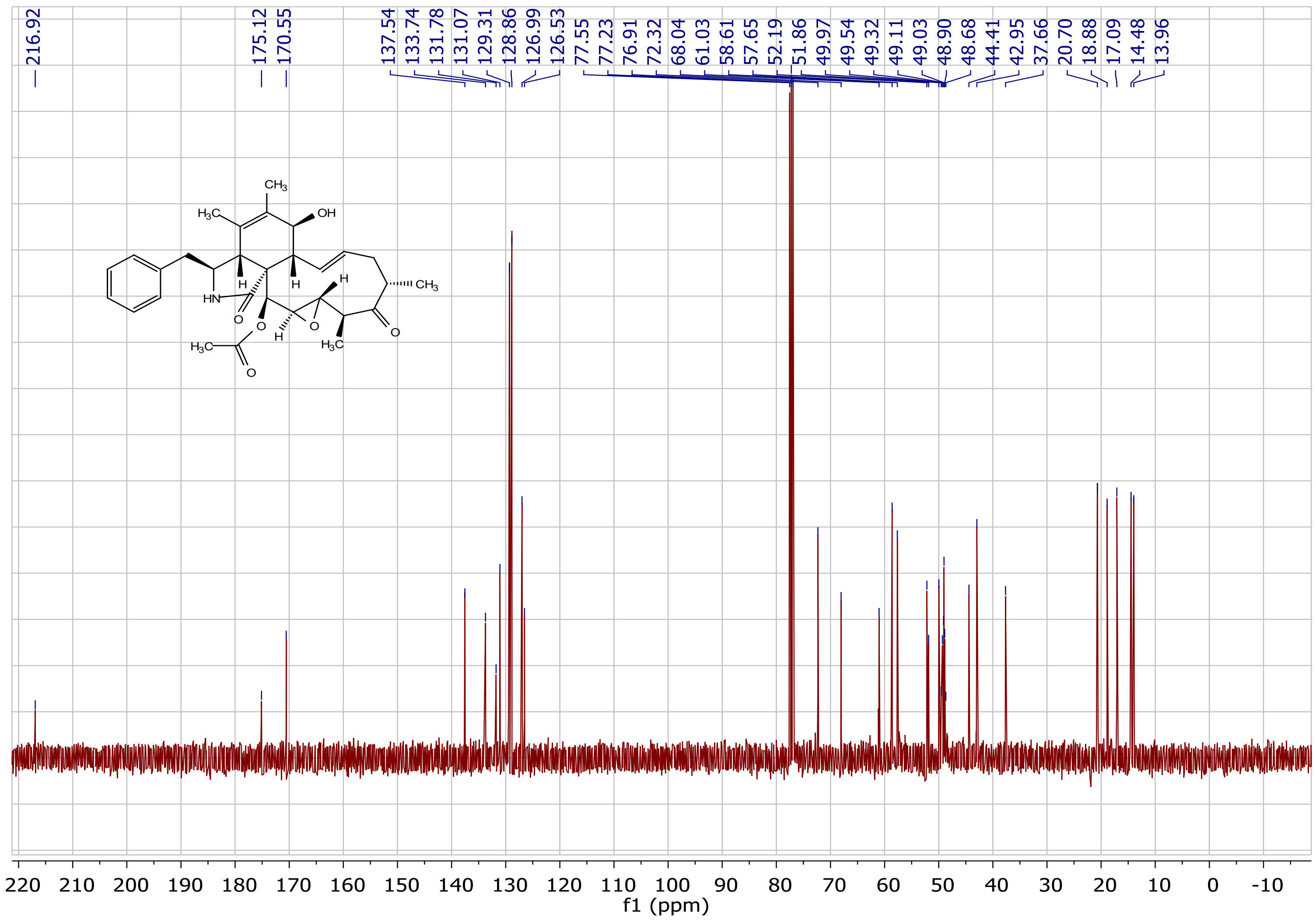

Figure S14: ^{13}C NMR Spectrum of compound **3**

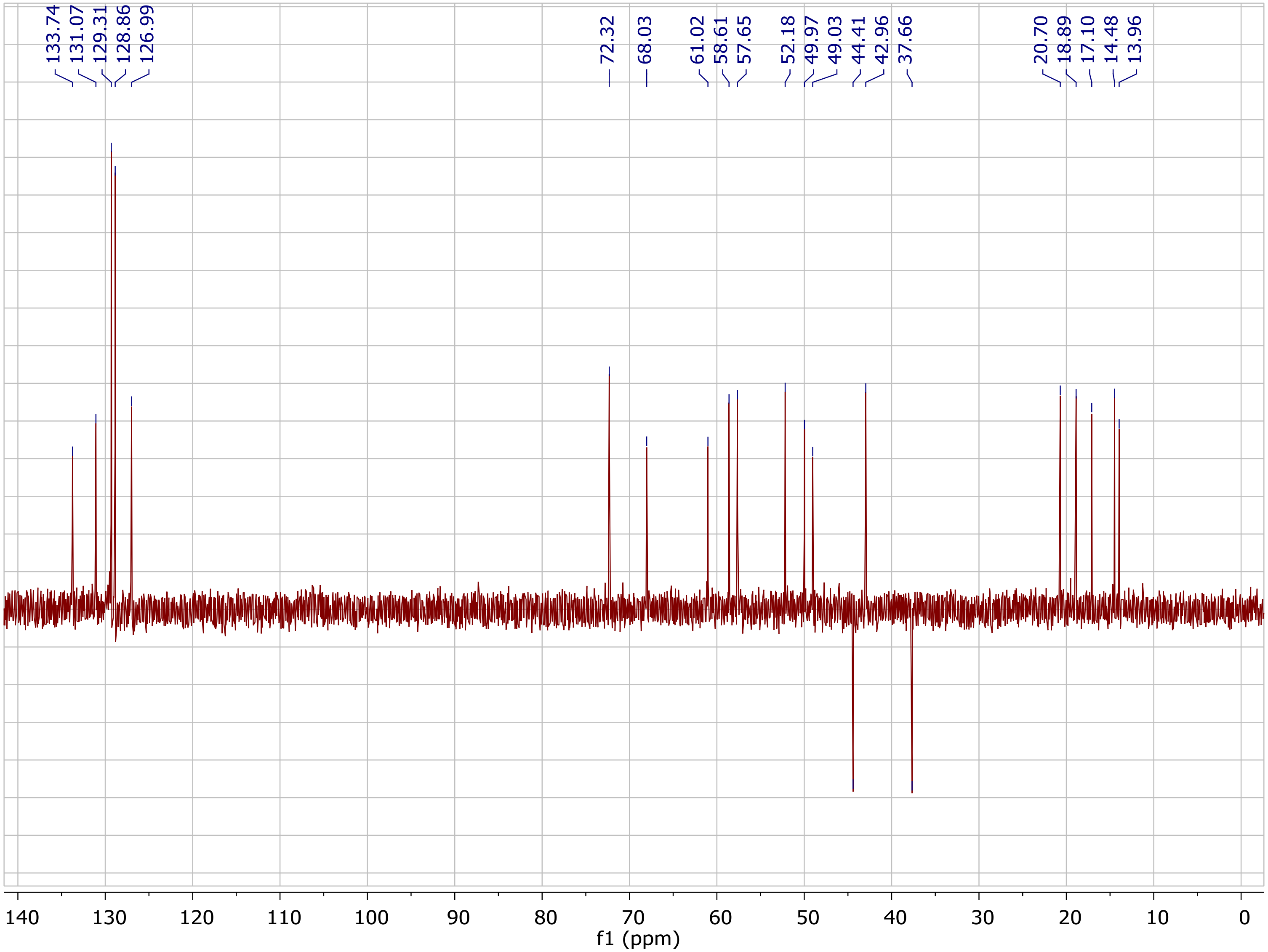

Figure S15: DEPT Spectrum of compound **3**

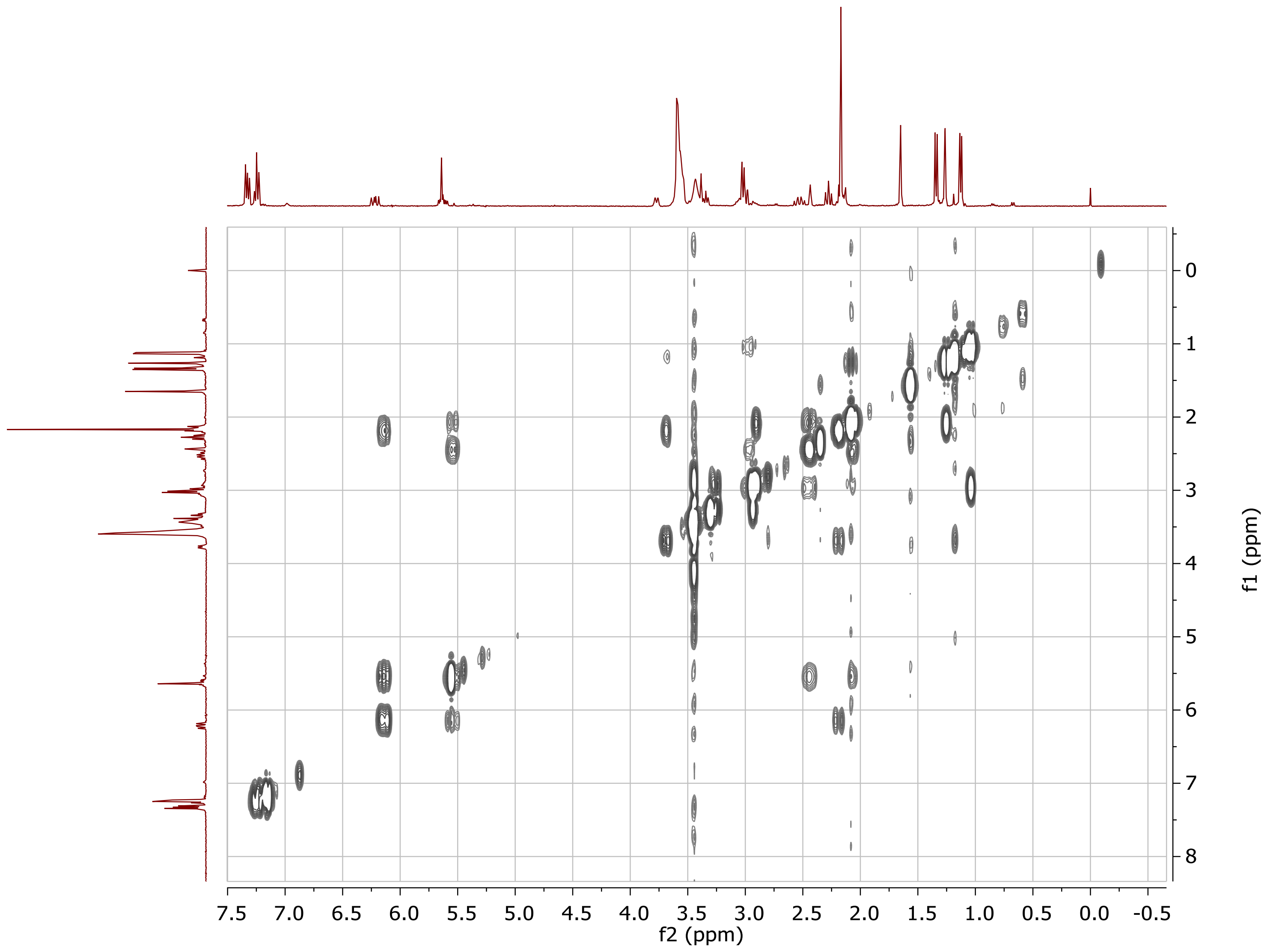

Figure S16: COSY Spectrum of compound **3**

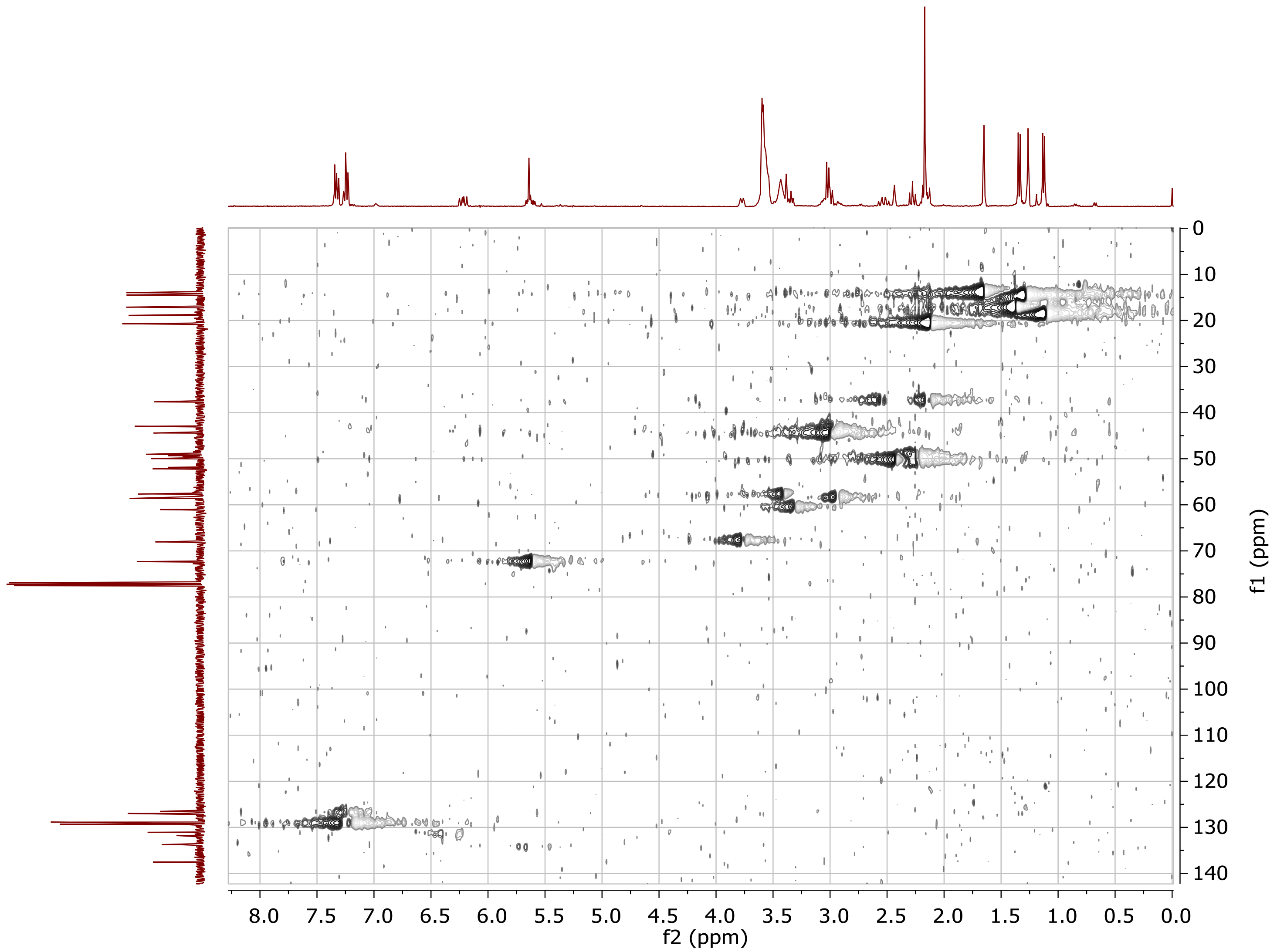

Figure S17: HMQC Spectrum of compound **3**

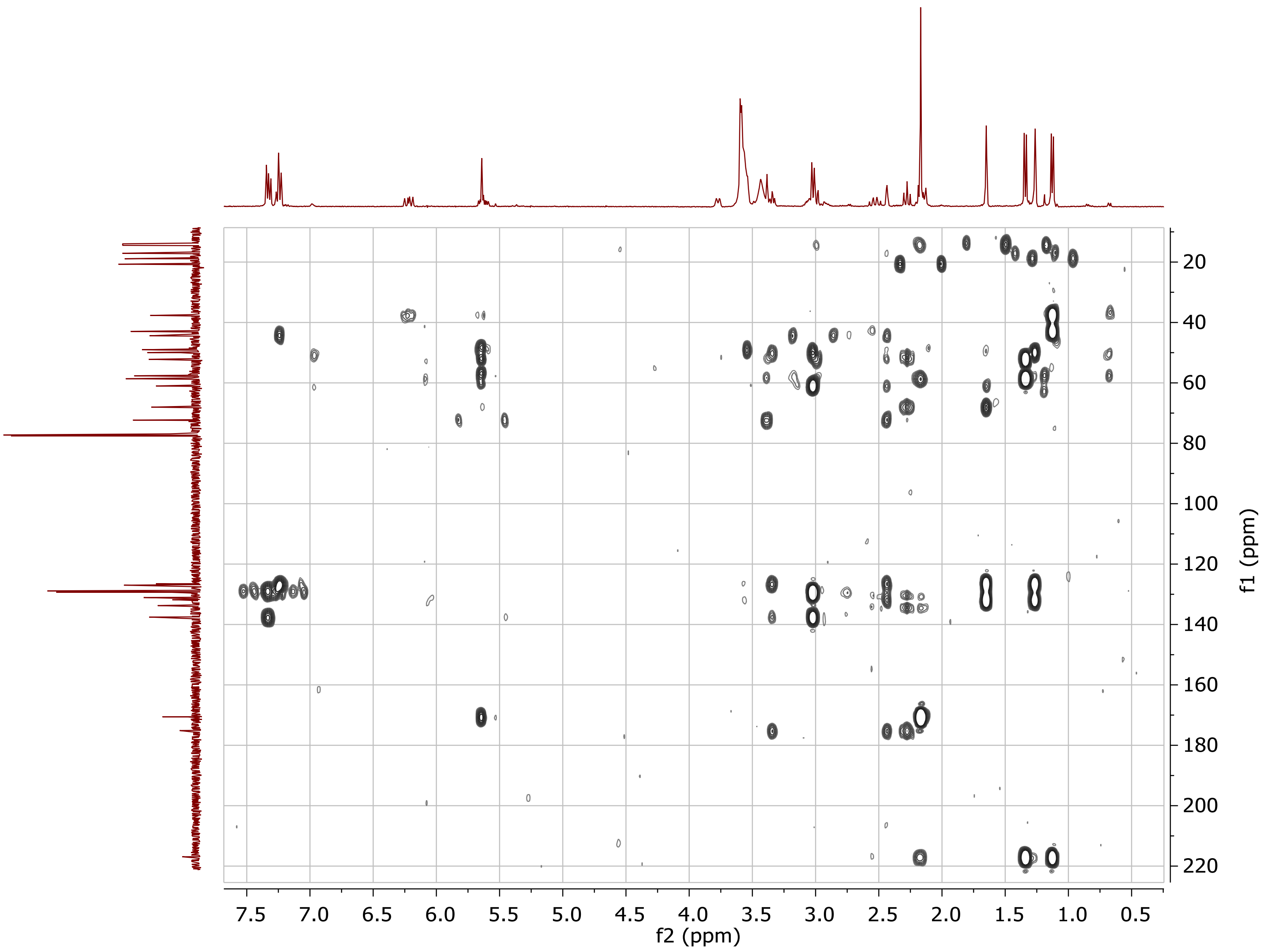

Figure S18: HMBC Spectrum of compound **3**

Figure S19: ITS sequence alignment of UM10M with sequences published in GenBank

```
                        *        20         *        40         *        60         *        80         *
UM10M       : TCCGTAGGTGAACCTGCGGAGGGATCATTACCGAGTTGTCCTCCGTG-ACACTCCCTTCATACCCCCTGTGAAC-ATACCATC--GTTGCCTC :  89
JQ761479.1  : ---------------------GGATCATTACCGAGTTGTCCTCCGTG-ACACTCCCTTCATACCCCCTGTGAAC-ATACCATC--GTTGCCTC :  68
KC477228.1  : ------------------------TCATTACCGAGTTGTCCT-CTGTGACACTCCCTTTACACCCCTTGTGAAC-ATACCGTC--GTTGCCTC :  65
KX037428.1  : ---------------------GGATCATTACTGAGTTGTCCTCCGAGGACACTCCCTTCACACCCCTTGTGAAC-ATACCGTC--GTTGCCTC :  69
KU683913.1  : ---------------------GGATCATTACTGAGTTGTCCTCCGAGGACACTCCCTTTACACCCCTTGTGAAC-ATACCGTC--GTTGCCTC :  69
AF201704.1  : TCGGTTGGTGAACCAGCGGAGGGATCATTACAGAGTTAC----CAAA---ACTCCC---AAACCCA-TGTGAAC-ATACC-TCGCGTTGCCTC :  80
DQ641634.1  : --------------------GGGATCATTACTGAGTTTT--GAAAAAAG-ACTCCC---AAACCC--TGTGAAC-CTACC-TATCGTTGCCTC :  63
EF026121.1  : TCCGTAGGTGAACCTGCGGAGGGATCATTAAAGAGTTTT----C-TACA-ACTCCC---AAACCCC-TGTGAAC-ATACCTTC-TGTTGCCTC :  81
EF026122.1  : TCCGTAGGTGAACCTGCGGAGGGATCATTAAAGAGTT-T----AAAACA-ACTCCT---AAACCCA-TGTGAAC-ATACCTTC-TGTTGCCTC :  81
GU292818.1  : TCCGTAGGTGAACCTGCGGAGGGATCATTAAAGAGAGCC----TAAAAA-ACTCCCGCAAACCCCA-TGTGAAC-ATACCAT-ACGTTGCCTC :  85
GU292822.2  : TCCGTAGGTGAACCTGCGGAGGGATCATTAAAGAGTTCT-------ATA-ACTCCC---AAACCCA-TGTGAAC-ATACCTT-ACGTTGCCTC :  79
JQ759872.1  : ---------------------GGATCATTACAGAGTTAT----CTAA---ACTCCC---AAACCCA-TGTGAAC-ATACC-TCGCGTTGCCTC :  59
JQ760608.1  : ---------------------GGATCATTACAGAGTTGT----CCTA---ACTCCC---AAACCCA-TGTGAAC-ATACCGTC-TGTTGCCTC :  59
JQ846087.1  : TCCGTTGGTGAACCAGCGGAGGGATCATTACTGAGTTTT--GAAAAAAG-ACTCCC---AAACCC--TGTGAAC-CTACC-TATCGTTGCCTC :  83
KC708575.1  : -------------------------------------------------------------------TGTGAAC-ATACCTTC-TGTTGCCTC :  24
KC845930.1  : ---------------------------------------------------------------------TGAAC-ATACCTTC-TGTTGCCTC :  22
KP133215.1  : TCCGTTGGTGAACCAGCGGAGGGATCATTAGAGAGTTCT----CATACA-ACTCCC---AAACCCA-TGTGAAC-ATACCTTC-TGTTGCCTC :  82
KP133218.1  : TCCGTTGGTGAACCAGCGGAGGGATCATTAAAGAGAGCC----TAAAAA-ACTCCCGTAAACCCCA-TGTGAAC-ATACCAT-ACGTTGCCTC :  85
KU683765.1  : ---------------------GGATCATTATAGAGTTAT----CTAA---ACTCCC---AAACCCA-TGTGAAC-ATACC-TCGCGTTGCCTC :  59
KU683999.1  : --------------------------------------------ATA---ACTCCC---AAACCCA-TGTGAAC-ATACC-TCATGTTGCCTC :  40
KU747773.1  : ----------------------------TACAGAGTTGT----C-AA---ACTCCC---AA-CCCA-TGTGAAC-CTACC-TCGCGTTGCCTC :  50
KU747908.1  : --------------------------------------------AAA---ACTCCC---AAACCCA-TGTGAAC-ATACC-TCTCGTTGCCTC :  40
KX774096.1  : --------------------------------------------------------------------GTGAAC-ATACC-TCATGTTGCCTC :  23
KX778681.1  : -------------------------------------------------------C---AA-CCCT-TTTTAA---TTACTTCATGTTGCCTC :  30
KY250390.1  : TCCGTTGGTGAACCAGCGGAGGGATCATTAAAGAGTTTT------TACA-ACTCCC---AAACCCC-TGTGAAC-ATACCTTC-TGTTGCCTC :  80
KY250391.1  : TC-GT-GGTGA--CAGCGGAGGGATCATTAAAGAGTTTT------TACA-ACTCCC---AAACCCC-TGTGAAC-ATACCTTC-TGTTGCCTC :  76
LN714575.1  : -----------------------ATCATTAAAGAGT-GT----AATA---ACTCCC---AAACCCA-TGTGAAC-ATACC-TCATGTTGCCTC :  56
MF153390.1  : ------------------------------AAGAGTTTT----C-TACA-ACTCCC---AAACCCC-TGTGA-C-GTACCTTC-TGTTGCCTC :  50
MF663581.1  : TCCGTTGGTGAACCAGCGGAGGGATCATTAGAGAGTCT------AAACA-ACTCCC---AAACCCACTGTGAAC-ATACC-CCACGTTGCCTC :  81
MG543928.1  : ----------------------------------------------AAA-ACTCCC---AAACCCA-TGTGAAC-ATACC-TCGCGTTGCCTC :  40
MG543932.1  : ----------------------------------------------AAA-ACTCCC---AAACCCA-TGTGAAC-ATACC-TCGCGTTGCCTC :  40
MG543935.1  : --------------------------------------------AAA---ACTCCC---AAACCCA-TGTGAAC-ATACC-TCGCGTTGCCTC :  40
MG543943.1  : ----------------------------------------------AAA-ACTCCC---AAACCCA-TGTGAAC-ATACC-TCGCGTTGCCTC :  40
MG543947.1  : ----------------------------------------------AAA-ACTCCC---AAACCCA-TGTGAAC-ATACC-TCGCGTTGCCTC :  40
MG832444.1  : TCCGTTGGTGAACCAGCGGAGGGATCATTAAAGAGTGT-------AATA-ACTCCC---AAACCCA-TGTGAAC-ATACC-TCATGTTGCCTC :  79
MH178723.1  : ---------------------------TTACAGAGTTAT----CAAA---ACTCCC---AAACCCA-TGTGAAC-ATACC-TCGCGTTGCCTC :  53
MH305505.1  : ---------------------------------------------------------------------------ATACCAT-ACGTTGCCTC :  17
MH931277.1  : TCCGTTGGTGAACCAGCGGAGGGATCATTACAGAGTTAT----CTAA---ACTCCC---AAACCCA-TGTGAAC-ATACC-TCGCGTTGCCTC :  80
MK192915.1  : --------------------------------------------------ACTCCC---AAACCCA-TGTGAAC-ATACC-TCTCGTTGCCTC :  37
MK367486.1  : ------------------------------GGGAATAGG----TGTATA-ACTCCC---AA-CCCA-TGTGAAC-ATACC-TCATGTTGCCTC :  51
MK367543.1  : --------------------GGGGGC-TTA-AGAG--------CTTCAA-ACTCCC---AA-CCCA-TGTGAAC-ATACC-TCGCGTTGCCTC :  55
NR_160210.1 : TCCGTTGGTGAACCAGCGGAGGGATCATTACAGAGTTCT----CGCA---ACTCCC---ACACCCT-TGTGAACCATACC-ACATGTTGCCTC :  81
```

Figure S19: ITS sequence alignment of UM10M with sequences published in GenBank

```
                     100         *        120         *        140         *        160         *        180
UM10M       : ----GG-----------------------------------------------------------------CGTG-----GCGCCCGCCGGTG : 108
JQ761479.1  : ----GG-----------------------------------------------------------------CGTG-----GCGCCCGCCGGTG :  87
KC477228.1  : GGTGGG-----------------------------------------------------------------CGCGTCACAACGCCCGCCGGTG :  93
KX037428.1  : GGTGGG-----------------------------------------------------------------CGCGTTACCACGCCCGCCGGCG :  97
KU683913.1  : GGTGGG-----------------------------------------------------------------CGCGTTACCACGCCCGCCGGCG :  97
AF201704.1  : GGCAGGTGG---CGT--CCTACCC----CGTGAGACC--TACCCTGTAGG-A--CCTACCCGG---TAG-GC--G-----AC-CCTGCCGACG : 147
DQ641634.1  : GGTAGGTGGCCTCAT----TGCAC----CGTGGGGATATCTCCCTATGG-------TATAGTG----GGTGC--------AC-CCTGCCGAAG : 128
EF026121.1  : GGCAGGCCT---CG---CCTACCCT---CGTAGCCCCC-TACACCGTAGG-G--CCTACGCCGGG-TGGTGCGCGG----AC-CCTGCCGGCG : 155
EF026122.1  : GGCAGGTCG---CG---CTTACCC----TGTAGCGCCT-TACCCTGTAAG-G--CCTACCCGGA--AGGCGCGGG---TAACCCTGCCGGCG : 155
GU292818.1  : GGCAGGCCT---CAT-ACGTGCCCC---C-TCATGGCG-TGCCCGGCGCGCAGCCCCGCAAGGGGGGGGGGCCGCGGGTGAGACCTGCCGGTG : 169
GU292822.2  : GGCAGGT-----CGT-GCCTACCC----CGTAGCGCCC-TACCCTGTAGG-A--CCTACCCGGT--AGACGCGGG----TAAGCCTGCCGGCG : 152
JQ759872.1  : GGCAGGTGG---CGT--CCTACCC----CGTGATACC--TACCCTGTAGG-A--CCTACCCGG---TAG-GC--G-----AC-CCTGCCGACG : 126
JQ760608.1  : GGCAGGTCA---AGG--CCCGCGT----AGCGTG-CC--AGCAC---------------------------------------CCTGCCGGCG : 101
JQ846087.1  : GGTAGGTGGCCTCAT----TGCAC----CGTGGGGATATCTCCCTATGG-------TATAGTG----GGTGC--------AC-CCTGCCGAAG : 148
KC708575.1  : GGCAGGCCT---CG---CCTACCCT---CGTAGCCCCC-TACACCGTAGG-G--CCTACGCCGGG-TGGTGCGCGG----AC-CCTGCCGGCG :  98
KC845930.1  : GGCAGGCCT---CG---CCTACCCT---CGTAGCCCCC-TACACCGTAGG-G--CCTACGCCGGG-TGGTGCGCGG----AC-CCTGCCGGCG :  96
KP133215.1  : GGCAGGTCGCGCCGGCGCCTACCG----CGTAGCACCC-TACCCCGTAGG-G--CCTACCC--GGGACGCGCTGGCGGTCCCCCTGCCGGCG : 165
KP133218.1  : GGCAGGCCT---CAT-ACGTGCCCC---CCTCATG-CG-TGCCCGGCGCGCAACCCCGCAAGGGGGGGGGGACGCGGGTGGGACCTGCCGGTG : 169
KU683765.1  : GGCAGGTGG---CGT--CCTACCC----CGTGATACC--TACCCTGTAGG-A--CCTACCCGG---TAG-GC--G-----AC-CCTGCCGACG : 126
KU683999.1  : GGCAGGTCG---CG---CCTCCCT----CGTAGGCCC--TACCCCGTAGG-CT-CCTACGCGG---AAG-GC--GCGGGTACCCCTGCCGGTG : 113
KU747773.1  : GGCAGGCGG---CGT--CCCACCC----TGGAAGACC--TACCCCGTAGG-AGGCCTATCCGGGGGCGG-GC--G-----AC-CCTGCCGAAG : 122
KU747908.1  : GGCGGGCGC---CGT--CCTACC-----------ACC--A--CCGGT-GG-A---------GG-----G-GC--G-----AAACCCGCCGACG :  89
KX774096.1  : GGCAGGT-----CGT-GCCT-CCCT---CGTAGGTCC--TACCCTGTAGGCT--CCTACCCGGA--AGGCACGGG----TACCCCTGCCGGTG :  96
KX778681.1  : GGCAGGT-----CGT-GCCT-CCCT---CGTAGGTCC--TACCCTGTAGG-CT-CCTACCCGGA--AGGCGCGGG----TACCCCTGCCGGTG : 103
KY250390.1  : GGCAGG-C---CCTG-GCCTACCC----TGTAGCGCCCCTACACTGTAGG-G--CCTGCTTTAGGGGGGCGCTGGGGGAACC---TGCCGGCG : 158
KY250391.1  : GGCAGG-C---CCTG-GCCTACCC----TGTAGCGCCCCTACATTGTAGG-G--CTTGCTTGAGGGGGGCGCTGGGGGAACC---TGCCGGCG : 154
LN714575.1  : GGCAGGTCG---CG---CCTCCCT----CGTAGGCCC--TACCCCGTAGG-CT-CCTACGCGG---AAG-GC--GCGGGTACCCCTGCCGGTG : 129
MF153390.1  : GGCAGGCCT---CG---CCTACCCT---CGTAGCCCCC-TACACCGTAGG-G--CCTACGC-GGG-GGGTGCGGGG----AC-CCTGCCGGCG : 123
MF663581.1  : GGCAGG------CGC--CCGGCTC----CGCGACCCCTCGGGGCTGCGGGGTGGC----------------------------CCTGCCGGCG : 134
MG543928.1  : GGCAGGTGG---CGT---TCGCCC----CGTAAGAACC-TACCCTGTAGGAA--CGTACCCGG---TAG-ACG-------AC-CCTGCCGACG : 108
MG543932.1  : GGCAGGTGG---CGT--CTCGCCC----CGTAAGAACC-TACCCTGTAGGAC--CTTACCCGG---TAG-ACG-------AC-CCTGCCGACG : 109
MG543935.1  : GGCAGGTGG---CGT--CCTACCC----CGTGAGACC--TACCCTGTAGG-A--CCTACCCGG---TAG-GC--G-----AC-CCTGCCGACG : 107
MG543943.1  : GGCAGGTGG---CGT--CTCGCCC----CGTAAGAACC-TACCCTGTAGGGT--CTTACCCGG---TAG-ACG-------AC-CCTGCCGACG : 109
MG543947.1  : GGCAGGTGG---CGT--CTCGCCC----CGTAAGAACC-TACCCTGTAGGAT--CTTACCCGG---TAG-ACG-------AC-CCTGCCGACG : 109
MG832444.1  : GGCAGGTCG---CG---CCT-CTCT---CGTAGGTCC--TACGCGGAAGG--------CGCGGG--------------TACCCCTGCCGGCG : 137
MH178723.1  : GGCAGGTGG---CGC--CCCGCCCT----GTGAGGGCC-TACCCTGTAGGAG--CTCACCCGG----TGGTCG--------CCCCTGCCGACG : 122
MH305505.1  : GGCAGGCCT---CAT-ACGTGCCCC---C-TCATGGCG-TGCCCGGCGCGCAGCCCCGCAAGGGGGGGGGGCCGCGGGTGAGACCTGCCGGTG : 101
MH931277.1  : GGCAGGTGG---CGT--CCTACCC----CGTGATACC--TACCCTGTAGG-A--CCTACCCGG---TAG-GC--G-----AC-CCTGCCGACG : 147
MK192915.1  : GGCGGGCGC---CGT--CCTACCCCTCGCGGGTGGTCGAAACCC------------------------------------------GCCGAAG :  83
MK367486.1  : GGCAGGT-----CGT-GCCT-CCCT---CGTAGGTCC--TACCCTGTAGG-CT-CCTACCCGGA--AGGCGCGGG----TACCCCTGCCGGTG : 124
MK367543.1  : GGCAGGTGG---CGT--CTCGCCC----CGTAAGGTGC-TACCCTGTAGGGT--CTTACCCGG---TAG-ACG-------AC-CCTGCCGACG : 124
NR_160210.1 : GGCGGGGCG---CGA--CC-ACCC----TGTGGCAACC-TACCCTGTAGGCG---CTACCCTGTG--GACGCGGC---TCACCCC-GCCGGCG : 154
```

Figure S19: ITS sequence alignment of UM10M with sequences published in GenBank

```
                         *        200         *        220         *        240         *        260         *        28
UM10M        : GCCCATTACTACC-AAATTCTGTACACTTTGAAAACTTGATTCTGA-----ACTT-----ATAACAAATTATGTTAAAA-CTTTCAACAACGG : 189
JQ761479.1   : GCCCATTACTACC-AAATTCTGTACATTTTGAAAAATTGACTCTGA-----ACTT-----ATAACAAATCATGTTAAAA-CTTTCAACAACGG : 168
KC477228.1   : GCCCATTTATAC-CAAATTCTGTTTACTTTGAAAATTTACTTCTGA-----ATAT-----ATAACAAATCATGTTAAAA-CTTTCAACAACGG : 174
KX037428.1   : GCCCCTT--TACCTAAATTCTGTTAATCTT-ACTACTT----CTGA-----ACCT-----ATAACAAATCATGTTAAAA-CTTTCAACAACGG : 172
KU683913.1   : GCCCCTT--TACCTAAATTCTGTTAATCTT-ACTACTT----CTGA-----ACCT-----ATAACAAATCATGTTAAAA-CTTTCAACAACGG : 172
AF201704.1   : GCCCCCG-AAACTCT-GTTTT--T-ATAGC--ATTGGA-CTTCTGA-----AAAG-----ATAACTAAATAAGTTAAAA-CTTTCAACAACGG : 221
DQ641634.1   : GCCCCTATAAACTCT-TTGTATTTTG-AAC--ATTGGATCTTCTGA-----GTGG-----AAAAAAAAATTAGTTAAAAACTTTCAACAACGG : 207
EF026121.1   : GCCCGCG-AAACTCT-GTTT-------AGC--ACTGAA-TCTCTGA-----ACAT-----ATAACTAAATAAGTTAAAA-CTTTCAACAACGG : 225
EF026122.1   : GCCCAAG-AAACTCT-GTTT-------AGC--ATTGAA--TTCTGA-----ATAT-----ATAACTAAATAAGTTAAAA-CTTTCAACAACGG : 224
GU292818.1   : GCCCAAGCAAACCCT-GTT--------ATC--ACTGAA--TTCTGAGCCC-GCAT-----GTAGGAAATTAAGTCAAAA-CTTTCAACAACGG : 242
GU292822.2   : GCCCACG-AAACTCT-GTTT-------AGT--ATTGAA--TTCTGA-----ACCT-----GTAACAAAATAAGTTAAAA-CTTTCAACAACGG : 221
JQ759872.1   : GCCCCCG-AAACTCT-GTTTT--T-ATAGC--ATTGGA-CTTCTGA-----AAAG-----ATAACTAAATAAGTTAAAA-CTTTCAACAACGG : 200
JQ760608.1   : GCCCACG-AAATACT-GTTTT-----CTTC---TTGAA--TTCTGAACTTGAAAC-----AAAAAAAAATAAGTTAAAA-CTTTCAACAACGG : 176
JQ846087.1   : GCCCCTATAAACTCT-TTGTATTTTG-AAC--ATTGGATCTTCTGA-----GTGG-----AAAAAAAAATTAGTTAAAAACTTTCAACAACGG : 227
KC708575.1   : GCCCGCG-AAACTCT-GTTT-------AGC--ACTGAA-TCTCTGA-----ACAT-----ATAACTAAATAAGTTAAAA-CTTTCAACAACGG : 168
KC845930.1   : GCCCGCG-AAACTCT-GTTT-------AGC--ACTGAA-TCTCTGA-----ACAT-----ATAACTAAATAAGTTAAAA-CTTTCAACAACGG : 166
KP133215.1   : GCCCACG-AAACTCT-GTTT-------AGCTTATTGAA--TTCTGA-----ACGT-----ATAACTAAATTAGTTAAAA-CTTTCAACAACGG : 236
KP133218.1   : GCCCAAGCAAACCCT-GTC--------GTC--ACTGAA--TTCTGAGCCC-GCAT-----GTAGGAAATTAAGTCAAAA-CTTTCAACAACGG : 242
KU683765.1   : GCCCCCG-AAACTCT-GTTTT--T-ATAGC--ATTGGA-CTTCTGA-----AAAG-----ATAACTAAATAAGTTAAAA-CTTTCAACAACGG : 200
KU683999.1   : GCCCACG-AAACTCT-GTCT------CATC--GTTGAA--TTCTGA-----ACCT-----ATAACTAAATAAGTTAAAA-CTTTCAACAACGG : 183
KU747773.1   : GCCCCCCCAAACTCT-GTCTT-CT-ACAGC--ATTGGA-CCTCTGA-----AAAG-----ATGACTAAATCAGTTAAAA-CTTTCAACAACGG : 198
KU747908.1   : GCCCCCG-AAACTCT-GTTTTTCT-ATAGC--ATTGGA-CCTCTGAAGC--AAAG-----ATAACTAAATCAGTTAAAA-CTTTCAACAACGG : 168
KX774096.1   : GCCCAGG-AAACTCT-GTCT------CATC--GTTGAA--TTCTGA-----ACCT-----ATAACTAAATAAGTTAAAA-CTTTCAACAACGG : 166
KX778681.1   : GCCCAGG-AAACTCT-GTCT------CATC--GTTGAA--TTCTGA-----ACCT-----ATAACTAAATAAGTTAAAA-CTTTCAACAACGG : 173
KY250390.1   : GCCCGCA-AAACTCT-GTTT-------AGC--ATTGAA--TTCTGA-----ACGT-----ATAACTAAATAAGTTAAAA-CTTTCAACAACGG : 227
KY250391.1   : GCCCGCA-AAACTCT-GTTT-------AGC--ATTGAA--TTCTGA-----ACGT-----ATAACTAAATAAGTTAAAA-CTTTCAACAACGG : 223
LN714575.1   : GCCCACG-AAACTCT-GTCT------CATC--GTTGAA--TTCTGA-----ACCT-----ATAACTAAATAAGTTAAAA-CTTTCAACAACGG : 199
MF153390.1   : GCCCGCA-AAACTCT-GTTT-------AGC--ACTGAA-TCTCTGA-----ACAT-----GCAACTAAATAAGTTAAAA-CTTTCAACAACGG : 193
MF663581.1   : GCCCACG-AAACTCTTGTCT-------AGC--ACTGAA--TTCTGAGCCCGAGAGGGATAAAAACAAAATTAGTTAAAA-CTTTCAACAACGG : 214
MG543928.1   : GCCCCCG-AAACTCT-GTTTT----ATAGC--ATTAAA-CTTCTGA-----AAAC-----ATAACTAAATAAGTTAAAA-CTTTCAACAACGG : 181
MG543932.1   : GCCCCCG-AAACTCT-GTTTT----ATAGC--ATTAAA-CTTCTGA-----AAAT-----ATAACTAAATAAGTTAAAA-CTTTCAACAACGG : 182
MG543935.1   : GCCCCCG-AAACTCT-GTTTT--T-ATAGC--ATTGGA-CTTCTGA-----AAAG-----ATAACTAAATAAGTTAAAA-CTTTCAACAACGG : 181
MG543943.1   : GCCCCCG-AAACTCT-GTTTT----ATAGC--ATTAAA-TTTCTGA-----AAAT-----ATAACTAAATAAGTTAAAA-CTTTCAACAACGG : 182
MG543947.1   : GCCCCCG-AAACTCT-GTTTT----ATAGC--ATTAAA-CTTCTGA-----AAAT-----ATAACTAAATAAGTTAAAA-CTTTCAACAACGG : 182
MG832444.1   : GCCCACG-AAACTCT-GTCT------CATC--GTTGAA--TTCTGA-----ACCT-----ATAACTAAATAAGTTAAAA-CTTTCAACAACGG : 207
MH178723.1   : GCCCACG-AAACTCT-GTTTTTTTTATAGC--ATTAGA-CCTCTGA-----AAAG-----ATAACTAAATAAGTTAAAA-CTTTCAACAACGG : 199
MH305505.1   : GCCCAAGCAAACCCT-GTT--------ATC--ACTGAA--TTCTGAGCCC-GCAT-----GTAGGAAATTAAGTCAAAA-CTTTCAACAACGG : 174
MH931277.1   : GCCCCCG-AAACTCT-GTTTT--T-ATAGC--ATTGGA-CTTCTGA-----AAAG-----ATAACTAAATAAGTTAAAA-CTTTCAACAACGG : 221
MK192915.1   : GCCCCCG-AAACTCT-GTTTTT-T-ATAGC--ATTAGA-CCTCTGAAA---AAAG-----ATAACTAAATCAGTTAAAA-CTTTCAACAACGG : 160
MK367486.1   : GCCCAGG-AAACTCT-GTCT------CATC--GTTGAA--TTCTGA-----ACCT-----ATAACTAAATAAGTTAAAA-CTTTCAACAACGG : 194
MK367543.1   : GCCCTCG-AAACTCT-GTTTTT---ATAAC--ATTAGA-CTTCTGA-----AAAG-----ATAACTAAATAAGTTAAAA-CTTTCAACAACGG : 198
NR_160210.1  : GCCCAC-CAAACTCT-GTTT--------ACT-TTTGAA--TTCTGA-----ACCT-----ATAACGAAATACGTTAAAA-CTTTCAACAACGG : 223
```

Figure S19: ITS sequence alignment of UM10M with sequences published in GenBank

```
                     0         *       300         *       320         *       340         *       360         *
UM10M        : ATCTCTTGGTTCTGGCATCGATGAAGAACGCAGCGAAATGCGATACGTAATGTGAATTGCAGAATTCAGTGAATCATCGAATCTTTGAACGCA : 282
JQ761479.1   : ATCTCTTGGTTCTGGCATCGATGAAGAACGCAGCGAAATGCGATACGTAATGTGAATTGCAGAATTCAGTGAATCATCGAATCTTTGAACGCA : 261
KC477228.1   : ATCTCTTGGTTCTGGCATCGATGAAGAACGCAGCGAAATGCGATACGTAATGTGAATTGCAGAATTCAGTGAATCATCGAATCTTTGAACGCA : 267
KX037428.1   : ATCTCTTGGTTCTGGCATCGATGAAGAACGCAGCGAAATGCGATACGTAATGTGAATTGCAGAATTCAGTGAATCATCGAATCTTTGAACGCA : 265
KU683913.1   : ATCTCTTGGTTCTGGCATCGATGAAGAACGCAGCGAAATGCGATACGTAATGTGAATTGCAGAATTCAGTGAATCATCGAATCTTTGAACGCA : 265
AF201704.1   : ATCTCTTGGTTCTGGCATCGATGAAGAACGCAGCGAAATGCGATAAGTAATGTGAATTGCAGAATTCAGTGAATCATCGAATCTTTGAACGCA : 314
DQ641634.1   : ATCTCTTGGTTCTGGCATCGATGAAGAACGCAGCGAAATGCGATACGTAATGTGAATTGCAGAATTTAGTGAATCATCGAATCTTTGAACGCA : 300
EF026121.1   : ATCTCTTGGTTCTGGCATCGATGAAGAACGCAGCGAAATGCGATAAGTAATGTGAATTGCAGAATTCAGTGAATCATCGAATCTTTGAACGCA : 318
EF026122.1   : ATCTCTTGGTTCTGGCATCGATGAAGAACGCAGCGAAATGCGATAAGTAATGTGAATTGCAGAATTCAGTGAATCATCGAATCTTTGAACGCA : 317
GU292818.1   : ATCTCTTGGTTCTGGCATCGATGAAGAACGCAGCGAAATGCGATAAGTAATGTGAATTGCAGAATTCAGTGAATCATCGAATCTTTGAACGCA : 335
GU292822.2   : ATCTCTTGGTTCTGGCATCGATGAAGAACGCAGCGAAATGCGATAAGTAATGTGAATTGCAGAATTCAGTGAATCATCGAATCTTTGAACGCA : 314
JQ759872.1   : ATCTCTTGGTTCTGGCATCGATGAAGAACGCAGCGAAATGCGATAAGTAATGTGAATTGCAGAATTCAGTGAATCATCGAATCTTTGAACGCA : 293
JQ760608.1   : ATCTCTTGGTTCTGGCATCGATGAAGAACGCAGCGAAATGCGATAAGTAATGTGAATTGCAGAATTCAGTGAATCATCGAATCTTTGAACGCA : 269
JQ846087.1   : ATCTCTTGGTTCTGGCATCGATGAAGAACGCAGCGAAATGCGATACGTAATGTGAATTGCAGAATTTAGTGAATCATCGAATCTTTGAACGCA : 320
KC708575.1   : ATCTCTTGGTTCTGGCATCGATGAAGAACGCAGCGAAATGCGATAAGTAATGTGAATTGCAGAATTCAGTGAATCATCGAATCTTTGAACGCA : 261
KC845930.1   : ATCTCTTGGTTCTGGCATCGATGAAGAACGCAGCGAAATGCGATAAGTAATGTGAATTGCAGAATTCAGTGAATCATCGAATCTTTGAACGCA : 259
KP133215.1   : ATCTCTTGGTTCTGGCATCGATGAAGAACGCAGCGAAATGCGATAAGTAATGTGAATTGCAGAATTCAGTGAATCATCGAATCTTTGAACGCA : 329
KP133218.1   : ATCTCTTGGTTCTGGCATCGATGAAGAACGCAGCGAAATGCGATAAGTAATGTGAATTGCAGAATTCAGTGAATCATCGAATCTTTGAACGCA : 335
KU683765.1   : ATCTCTTGGTTCTGGCATCGATGAAGAACGCAGCGAAATGCGATAAGTAATGTGAATTGCAGAATTCAGTGAATCATCGAATCTTTGAACGCA : 293
KU683999.1   : ATCTCTTGGTTCTGGCATCGATGAAGAACGCAGCGAAATGCGATAAGTAATGTGAATTGCAGAATTCAGTGAATCATCGAATCTTTGAACGCA : 276
KU747773.1   : ATCTCTTGGTTCTGGCATCGATGAAGAACGCAGCGAAATGCGATAAGTAATGTGAATTGCAGAATTCAGTGAATCATCGAATCTTTGAACGCA : 291
KU747908.1   : ATCTCTTGGTTCTGGCATCGATGAAGAACGCAGCGAAATGCGATAAGTAATGTGAATTGCAGAATTCAGTGAATCATCGAATCTTTGAACGCA : 261
KX774096.1   : ATCTCTTGGTTCTGGCATCGATGAAGAACGCAGCGAAATGCGATAAGTAATGTGAATTGCAGAATTCAGTGAATCATCGAATCTTTGAACGCA : 259
KX778681.1   : ATCTCTTGGTTCTGGCATCGATGAAGAACGCAGCGAAATGCGATAAGTAATGTGAATTGCAGAATTCAGTGAATCATCGAATCTTTGAACGCA : 266
KY250390.1   : ATCTCTTGGTTCTGGCATCGATGAAGAACGCAGCGAAATGCGATAAGTAATGTGAATTGCAGAATTCAGTGAATCATCGAATCTTTGAACGCA : 320
KY250391.1   : ATCTCTTGGTTCTGGCATCGATGAAGAACGCAGCGAAATGCGATAAGTAATGTGAATTGCAGAATTCAGTGAATCATCGAATCTTTGAACGCA : 316
LN714575.1   : ATCTCTTGGTTCTGGCATCGATGAAGAACGCAGCGAAATGCGATAAGTAATGTGAATTGCAGAATTCAGTGAATCATCGAATCTTTGAACGCA : 292
MF153390.1   : ATCTCTTGGTTCTGGCATCGATGAAGAACGCAGCGAAATGCGATAAGTAATGTGAATTGCAGAATTCAGTGAATCATCGAATCTTTGAACGCA : 286
MF663581.1   : ATCTCTTGGTTCTGGCATCGATGAAGAACGCAGCGAAATGCGATAAGTAATGTGAATTGCAGAATTCAGTGAATCATCGAATCTTTGAACGCA : 307
MG543928.1   : ATCTCTTGGTTCTGGCATCGATGAAGAACGCAGCGAAATGCGATAAGTAATGTGAATTGCAGAATTCAGTGAATCATCGAATCTTTGAACGCA : 274
MG543932.1   : ATCTCTTGGTTCTGGCATCGATGAAGAACGCAGCGAAATGCGATAAGTAATGTGAATTGCAGAATTCAGTGAATCATCGAATCTTTGAACGCA : 275
MG543935.1   : ATCTCTTGGTTCTGGCATCGATGAAGAACGCAGCGAAATGCGATAAGTAATGTGAATTGCAGAATTCAGTGAATCATCGAATCTTTGAACGCA : 274
MG543943.1   : ATCTCTTGGTTCTGGCATCGATGAAGAACGCAGCGAAATGCGATAAGTAATGTGAATTGCAGAATTCAGTGAATCATCGAATCTTTGAACGCA : 275
MG543947.1   : ATCTCTTGGTTCTGGCATCGATGAAGAACGCAGCGAAATGCGATAAGTAATGTGAATTGCAGAATTCAGTGAATCATCGAATCTTTGAACGCA : 275
MG832444.1   : ATCTCTTGGTTCTGGCATCGATGAAGAACGCAGCGAAATGCGATAAGTAATGTGAATTGCAGAATTCAGTGAATCATCGAATCTTTGAACGCA : 300
MH178723.1   : ATCTCTTGGTTCTGGCATCGATGAAGAACGCAGCGAAATGCGATAAGTAATGTGAATTGCAGAATTCAGTGAATCATCGAATCTTTGAACGCA : 292
MH305505.1   : ATCTCTTGGTTCTGGCATCTATGAAGAACGCAGCGAAATGCGATAAGTAATGTGAATTGCAGAATTCAGTGAATCATCGAATCTTTGAACGCA : 267
MH931277.1   : ATCTCTTGGTTCTGGCATCGATGAAGAACGCAGCGAAATGCGATAAGTAATGTGAATTGCAGAATTCAGTGAATCATCGAATCTTTGAACGCA : 314
MK192915.1   : ATCTCTTGGTTCTGGCATCGATGAAGAACGCAGCGAAATGCGATAAGTAATGTGAATTGCAGAATTCAGTGAATCATCGAATCTTTGAACGCA : 253
MK367486.1   : ATCTCTTGGTTCTGGCATCGATGAAGAACGCAGCGAAATGCGATAAGTAATGTGAATTGCAGAATTCAGTGAATCATCGAATCTTTGAACGCA : 287
MK367543.1   : ATCTCTTGGTTCTGGCATCGATGAAGAACGCAGCGAAATGCGATAAGTAATGTGAATTGCAGAATTCAGTGAATCATCGAATCTTTGAACGCA : 291
NR_160210.1  : ATCTCTTGGTTCTGGCATCGATGAAGAACGCAGCGAAATGCGATAAGTAATGTGAATTGCAGAATTCAGTGAATCATCGAATCTTTGAACGCA : 316
```

Figure S19: ITS sequence alignment of UM10M with sequences published in GenBank

```
                       380         *       400         *       420         *       440         *       460
UM10M        : CATTGCGCCCGCTGGTATTCCAGCGGGCATGCCTGTTCGAGCGTCATTTCAACCC-TTAAGCCCTCGTTGCTTAGTGTTGGGAGA-CTACCGC : 373
JQ761479.1   : CATTGCGCCCGCTGGTATTCCAGCGGGCATGCCTGTTCGAGCGTCATTTCAACCC-TTAAGCCCTCGTTGCTTAGTGTTGGGAGA-CTACC-C : 351
KC477228.1   : CATTGCGCCCGCTGGTATTCCAGCGGGCATGCCTGTTCGAGCGTCATTTCAACCC-TTAAGCCCTTGTTGCTTAGCGTTGGGAGATCACCCCG : 359
KX037428.1   : CATTGCGCCCGCTGGTATTCCAGCGGGCATGCCTGTTCGAGCGTCATTTCAACCC-TTAAGCCCCTGCTGCTTAGTGTTGGGAGC-CTACC-C : 355
KU683913.1   : CATTGCGCCCGCTGGTATTCCAGCGGGCATGCCTGTTCGAGCGTCATTTCAACCC-TTAAGCCCCTGTTGCTTAGTGTTGGGAGA-CTACC-G : 355
AF201704.1   : CATTGCGCCCACTAGTATTCTGGTGGGCATGCCTGTTCGAGCGTCATTTCAACCC-TTAAGCCCCTGTTGCTTAGCGTTGGGAGC-CTACTGG : 405
DQ641634.1   : CATTGCGCCCGCCAGTATTCTGGCGGGCATGCCTGTTCGAGCGTCATTTCAACCC-TTAAGCCTCTGTCGCTTAGTGTTGGGAGA-CTGCTAG : 391
EF026121.1   : CATTGCGCCCATTAGTATTCTAGTGGGCATGCCTGTTCGAGCGTCATTTCAACCC-TTAAGCCCCTGTTGCTTAGCGTTGGGAGC-CTACGG- : 408
EF026122.1   : CATTGCGCCCATTAGTATTCTAGTGGGCATGCCTGTTCGAGCGTCATTTCAACCC-TTAAGCC-TTGCTGCTTAGCGTTGGGAGC-CTACAG- : 406
GU292818.1   : CATTGCGCCCGCCAGTATTCTGGCGGGCATGCCTGTTCGAGCGTCATTTCAACCCCTTAAGCCCCTGTTGCTTAGCGTTGGGAGC-CTACGG- : 426
GU292822.2   : CATTGTGCCCACTAGTATTCTAGTGGGCATGCCTGTTCGAGCGTCATTTCAACCC-TTAAGCCTCTGTTGCTTAGTGTTGGGAGC-CTACGG- : 404
JQ759872.1   : CATTGCGCCCACTAGTATTCTGGTGGGCATGCCTGTTCGAGCGTCATTTCAACCC-TTAAGCCCCCGTTGCTTAGTGTTGGGAGC-CTACTGG : 384
JQ760608.1   : CATTGCGCCCATTAGCATTCTAGTGGGCATGCCTGTTCGAGCGTCATTTCAACCCCTTAAGCCTCTGTCGCTTAGTGTTGGGAGC-CTAC-GG : 360
JQ846087.1   : CATTGCGCCCGCCAGTATTCTGGCGGGCATGCCTGTTCGAGCGTCATTTCAACCC-TTAAGCCTCTGTCGCTTAGTGTTGGGAGA-CTGCTAG : 411
KC708575.1   : CATTGCGCCCATTAGTATTCTAGTGGGCATGCCTGTTCGAGCGTCATTTCAACCC-TTAAGCCCCTGTTGCTTAGCGTTGGGAGC-CTACGG- : 351
KC845930.1   : CATTGCGCCCATTAGTATTCTAGTGGGCATGCCTGTTCGAGCGTCATTTCAACCC-TTAAGCCCCTGTTGCTTAGCGTTGGGAGC-CTACGG- : 349
KP133215.1   : CATTGCGCCCATTAGTATTCTAGTGGGCATGCCTGTTCGAGCGTCATTTCAACCC-TCAAGCCCCCGTTGCTTGGTGTTGGGAGC-CTACGGG : 420
KP133218.1   : CATTGCGCCCGCCAGTATTCTGGCGGGCATGCCTGTTCGAGCGTCATTTCAACCCCTTAAGCCCCTGTTGCTTAGCGTTGGGAGC-CTACGG- : 426
KU683765.1   : CATTGCGCCCACTAGTATTCTGGTGGGCATGCCTGTTCGAGCGTCATTTCAACCC-TTAAGCCCCCGTTGCTTAGTGTTGGGAGC-CTACTGG : 384
KU683999.1   : CATTGCGCCCATTAGTATTCTAGTGGGCATGCCTGTTCGAGCGTCATTTCAACCC-TTAAGCCCTTGTTGCTTAGCGTTGGGAGC-CTAC-GG : 366
KU747773.1   : CATTGCGCCCGCCAGCATTCTGGCGGGCATGCCTGTTCGAGCGTCATTTCAACCC-TTAAGCCCT-GTTGCTTAGCGTTGGGAGC-CTACCGG : 381
KU747908.1   : CATTGCGCCCACCAGTATTCTGGTGGGCATGCCTGTTCGAGCGTCATTTCAACCC-TTAAGCCCCAGCTGCTTAGCGTTGGGAGC-CTACCGG : 352
KX774096.1   : CATTGCGCCCATTAGTATTCTAGTGGGCATGCCTGTTCGAGCGTCATTTCAACCC-TTAAGCCCTTGTTGCTTAGCGTTGGGAGC-CTACGG- : 349
KX778681.1   : CATTGCGCCCATTAGTATTCTAGTGGGCATGCCTGTTCGAGCGTCATTTCAACCC-TTAAGCCCTTGTTGCTTAGCGTTGGGAGC-CTACGG- : 356
KY250390.1   : CATTGCGCCCATTAGTATTCTAGTGGGCATGCCTGTTCGAGCGTCATTTCAACCC-TTAAGCCCCTGTTGCTTAGCGTTGGGAGC-CTACGG- : 410
KY250391.1   : CATTGCGCCCATTAGTATTCTAGTGGGCATGCCTGTTCGAGCGTCATTTCAACCC-TTAAGCCCCTGTTGCTTAGCGTTGGGAGC-CTACGG- : 406
LN714575.1   : CATTGCGCCCATTAGTATTCTAGTGGGCATGCCTGTTCGAGCGTCATTTCAACCC-TTAAGCCCTTGTTGCTTAGCGTTGGGAGC-CTAC-GG : 382
MF153390.1   : CATTGCGCCCATTAGTATTCTAGTGGGCATGCCTGTTCGAGCGTCATTTCAACCC-TTAAGCCCCTGTTGCTTAGCGTTGGGAGC-CTACGG- : 376
MF663581.1   : CATTGCGCCCGCCAGTATTCTGGCGGGCATGCCTGTCCGAGCGTCATTTCAAACCCTCAAGCCTC-GTTGCTTGGTGTTGGGAGC-CTACGG- : 397
MG543928.1   : CATTGCGCCCACTAGTATTCTGGTGGGCATGCCTGTTCGAGCGTCATTTCAACCC-TTAAGCCCCTGTTGCTTAGCGTTAGGAGC-CTACCGG : 365
MG543932.1   : CATTGCGCCCACTAGTATTCTGGTGGGCATGCCTGTTCGAGCGTCATTTCAACCC-TTAAGCCCCTGTTGCTTAGCGTTAGGAGC-CTACCGG : 366
MG543935.1   : CATTGCGCCCACTAGTATTCTGGTGGGCATGCCTGTTCGAGCGTCATTTCAACCC-TTAAGCCCCTGTTGCTTAGCGTTGGGAGC-CTACTGG : 365
MG543943.1   : CATTGCGCCCACTAGTATTCTGGTGGGCATGCCTGTTCGAGCGTCATTTCAACCC-TTAAGCCCCTGTTGCTTAGCGTTAAGAGC-CTACCGG : 366
MG543947.1   : CATTGCGCCCACTAGTATTCTGGTGGGCATGCCTGTTCGAGCGTCATTTCAACCC-TTAAGCCCCTGTTGCTTAGCGTTAGGAGC-CTACCGG : 366
MG832444.1   : CATTGCGCCCATTAGTATTCTAGTGGGCATGCCTGTTCGAGCGTCATTTCAACCC-TTAAGCCCTCGTTGCTTAGCGTTGGGAGC-CTACGG- : 390
MH178723.1   : CATTGCGCCCACCAGTATTCTGGTGGGCATGCCTGTTCGAGCGTCATTTCAACCC-TTAAGCCCCTGTTGCTTAGCGTTGGGAGC-CTACCGG : 383
MH305505.1   : CATTGCGCCCGCCAGTATTCTGGCGGGCATGCCTGTTCGAGCGTCATTTCAACCCCTTAAGCCCCTGTTGCTTAGCGTTGGGAGC-CTACGG- : 358
MH931277.1   : CATTGCGCCCACTAGTATTCTGGTGGGCATGCCTGTTCGAGCGTCATTTCAACCC-TTAAGCCCCCGTTGCTTAGTGTTGGGAGC-CTACTGG : 405
MK192915.1   : CATTGCGCCCACCAGTATTCTGGTGGGCATGCCTGTTCGAGCGTCATTTCAACCC-TTAAGCCCCAGTTGCTTAGCGTTGGGAGC-CTACCGG : 344
MK367486.1   : CATTGCGCCCATTAGTATTCTAGTGGGCATGCCTGTTCGAGCGTCATTTCAACCC-TTAAGCCCTTGTTGCTTAGCGTTGGGAGC-CTACGG- : 377
MK367543.1   : CATTGCGCCCACCAGTATTCTGGTGGGCATGCCTGTTCGAGCGTCATTTCAACCC-TTAAGCCCCTGTTGCTTAGCGTTGGGAGC-CTACTGG : 382
NR_160210.1  : CATTGCGCCCACTGGTATTCCGGTGGGCATGCCTGTTCGAGCGTCATTACAACAC-TTAAGCCC-TGTTGCTTAGCGTTGGGAGC-CTACGA- : 405
```

Figure S19: ITS sequence alignment of UM10M with sequences published in GenBank

```
                       *       480         *       500         *       520         *       540         *         5
UM10M       : CGCCATCCATGGCGGCGGCGGTAGCTCCCTAAAGCGAGTGGCG-GAGCCGGTCCCGTGCTCTAGGCGTAGTAGAT--TTT-AT------CTCG : 456
JQ761479.1  : CGCC----ATGGCGGCGGCGGTAGCTCCCTAAAGCGAGTGGCG-GAGCCGGTCCCGTGCTCTAGGCGTAGTAGAT--TTT-AT------CTCG : 430
KC477228.1  : CACC--------CCGCGGCGGTAGCTCCCTAAAGCGAGTAGCG-GAGCCGGTCCG-TGCTCTAGGCGTAGTAGAT--TTTCGT------CTCG : 434
KX037428.1  : CGCC----ATGCCGCCCGCGGTAGCTCCCCAAAGCGAGTGGCG-GAGCCGG-CCCGTGCTCTAGGCGTAGTAGAT--TTTCAT------CTCG : 434
KU683913.1  : CGCC----ATGCCGCG-GCGGTAGCTCCCTAAAGCGAGTGGCG-GAGCCGA-CCTGTGCTCTAGGCGTAGTAGAT--TTTCGT------CTCG : 433
AF201704.1  : AACT--------CTCCT---GTAGCTCTCCAAAGTCAGTGGCG-GAGCCGGTTCG-CACTCCAGACGTAGTAGCTT-TTACAT------ATCG : 478
DQ641634.1  : GATTT-------TTCCTT--ACAGCTCCCTAAATTCAGTGGCG-GAGTAGGTTCA-TGCCCCAGACGTAGTAGCTA-TTACA--------TCG : 464
EF026121.1  : ------------CAGC----GTAGCTCCCCAAAGTTAGTGGCG-TGGTCGGTTCA-CACTCCAGACGTAGTAGAT--TTTCGT------CTCG : 475
EF026122.1  : ------------CT------GTAGCTCCCCAAAGTTAGTGGCG-GAGTCGGTTCA-CACTCTAGACGTAGTAATT--TTTCAC------CTCG : 471
GU292818.1  : --CC--------CGCC----GTAGCTCCTCAAAGTTAGTGGCG-GAGTCGGCCCG-TACCCCAGGCGTAGTAGTTCATT-CAG------CTCG : 496
GU292822.2  : ---T--------CATA----GTAGCTCCTCAAAGTTAGTGGCG-GAGCTGGCTCA-CACTCCAGGCGTAGTAGATGTTATC---------TCG : 471
JQ759872.1  : AACT--------CTCCT---GTAGCTCCCCAAAGTCAGTGGCG-GAGCCGGTTCG-CACTCCAGACGTAGTAGCTT-TTACAC------ATCG : 457
JQ760608.1  : --CG--------TGCC----GTAGCTCCTTAAAGTTAGTGGCG-GAGTCGGTTCA-CACCCTAGACGTAGTAGGTTTTTACGC------ATCG : 431
JQ846087.1  : GATTT-------TTCCTT--ACAGCTCCCTAAATTCAGTGGCG-GAGTAGGTTCA-TGCCCCAGACGTAGTAGCTA-TTACA--------TCG : 484
KC708575.1  : ------------CAGC----GTAGCTCCCCAAAGTTAGTGGCG-TGGTCGGTTCA-CACTCCAGACGTAGTAGAT--TTTCGT------CTCG : 418
KC845930.1  : ------------CAGC----GTAGCTCCCCAAAGTTAGTGGCG-TGGTCGGTTCA-CACTCCAGACGTAGTAGAT--TTTCGT------CTCG : 416
KP133215.1  : GTCA--------CCC-----GTAGCTCCTCAAAGTTAGTGGCG-GAGTCGGTTCA-CACTCTAGACGTAGTAGAT--TTTCAC------CTCG : 490
KP133218.1  : --CC--------CGCC----GTAGCTCCTCAAAGTTAGTGGCG-GAGTCGGCCCG-TACCCCAGGCGTAGTAGTTCATT-CAG------CTCG : 496
KU683765.1  : AACT--------CTCCT---GTAGCTCCCCAAAGTCAGTGGCG-GAGCCGGTTCG-CACTCCAGACGTAGTAGCTT-TTACAC------ATCG : 457
KU683999.1  : ------------CACC----GTAGCTCCCCAAAGTCAGTGGCG-GAGCCGGCTCA-CACTCTAGACGTAGTAATTACT--CAC------CTCG : 433
KU747773.1  : GAGC--------CCCCG---GTAGCTCCCCAAAGTCAGTGGCG-GAGCCGGCTCG-CGCCCCAGACGTAGTAGCCCCTTACAC------GTCG : 455
KU747908.1  : AGAC--------CTCCTCCCGTAGCTCCCCAAAGTCAGTGGCG-GAGCCGGTTTAACACTCCAGACGTAGTAGCTC-ATACAC------ATCG : 429
KX774096.1  : ------------CACC----GTAGCTCCCCAAAGTCAGTGGCG-GAGCCGGCTCA-CACTCTAGACGTAGTAATTTCT--CAC------CTCG : 416
KX778681.1  : ------------CACC----GTAGCTCCCCAAAGTCAGTGGCG-GAGCCGGCTCA-CACTCTAGACGTAGTAATTTCT--CAC------CTCG : 423
KY250390.1  : ---A--------AGC-----GTAGCTCCCTAAAGTTAGTGGCG-TGGTCGGTTCA-CACTCCAGACGTAGTAGAT--TTTCAC------CTCG : 477
KY250391.1  : ---A--------AGC-----GTAGCTCCCTAAAGTTAGTGGCG-TGGTCGGTTCA-CACTCCAGACGTAGTAGAT--TTTCAC------CTCG : 473
LN714575.1  : ------------CACC----GTAGCTCCCCAAAGTCAGTGGCG-GAGCCGGCTCA-CACTCTAGACGTAGTAATTACT--CAC------CTCG : 449
MF153390.1  : ------------CAGC----GTAGCTCCCCAAAGTTAGTGGCG-TGGTCGGTTCA-CACTCCAGACGTAGTAGAT--TTTCGT------CTCG : 443
MF663581.1  : ------------CT------GTAGCTCCTCAAAGTCAGTGGCGTGGGCTGGCTCG-CACCCCAGATGTAGTAGTTA-TTTCTC-------TCA : 463
MG543928.1  : AACT--------C-TCTG--GTAGCTCCCCAAAGTCAGTGGCG-GAGCCGGTTCG-CACTCCAGACGTAGTAGCTT-TTACAC------GTCG : 438
MG543932.1  : AACT--------C-TCTG--GTAGCTCCCCAAAGTCAGTGGCG-GAGCCGGTTCG-CACTCCAGACGTAGTAGCTT-TTACAT------GTCG : 439
MG543935.1  : AACT--------CTCCT---GTAGCTCTCCAAAGTCAGTGGCG-GAGCCGGTTCG-CACTCCAGACGTAGTAGCTT-TTACAT------ATCG : 438
MG543943.1  : AACT--------C-TCTG--GTAGCTCCCCAAAGTCAGTGGCG-GAGCCGGTTCG-CACTCCAGACGTAGTAGCTT-TTACAC------GTCG : 439
MG543947.1  : AACT--------C-TCTG--GTAGCTCCCCAAAGTCAGTGGCG-GAGCCGGTTCG-CACTCCAGACGTAGTAGCTT-TTACAC------GTCG : 439
MG832444.1  : ------------CACC----GTAGCTCCCCAAAGTCAGTGGCG-GAGTCGGCTCA-CACTCTAGACGTAGTAATTTCT--CAC------CTCG : 457
MH178723.1  : AAAC--------CGTCCG--GTAGCTCCCCAAAGTCAGTGGCG-GAGCCGGTTCG-CACTCCAGACGTAGTAGCTT-TTACAC------GTCG : 457
MH305505.1  : --CC--------CGCC----GTAGCTCCTCAAAGTTAGTGGCG-GAGTCGGCCCG-TACCCCAGGCGTAGTAGTTCATT-CAG------CTCG : 428
MH931277.1  : AACT--------CTCCT---GTAGCTCCCCAAAGTCAGTGGCG-GAGCCGGTTCG-CACTCCAGACGTAGTAGCTT-TTACAC------ATCG : 478
MK192915.1  : ACCC--------CGTCCC--GTAGCTCCCCAAAGTCAGTGGCG-GAGCCGGCTTGACACTCCAGACGTAGTAGCTT-TTACAC------GTCG : 419
MK367486.1  : ------------CACC----GTAGCTCCCCAAAGTCAGTGGCG-GAGCCGGCTCA-CACTCTAGACGTAGTAATTTCT--CAC------CTCG : 444
MK367543.1  : ACCT--------TGTCCG--GTAGCTCCCCAAAGTCAGTGGCG-GAGCCGGTTCG-CACTCCAGACGTAGTAGCTT-TTACAC------GTCG : 456
NR_160210.1 : --CG--------TACC----GTAGCTCCTCAAAGTTAGTGGCG-GAGCCGGTCCG-TGCTCTAGGCGTAGTAGAT--TTTCGT------CTCG : 474
```

Figure S19: ITS sequence alignment of UM10M with sequences published in GenBank

```
                 60        *         580       *         600       *         620       *         640       *
UM10M        : CCTCCGTA-GCCGGATCTGGTTTTTCCTGCCATAAAA-CGAAGATT-TTTCTTCA----CTT----CTTTTAAGGTTGACCTCGGATCAGGTA : 538
JQ761479.1   : CCTCTGTA-GCCGGATCTGGTTTTTCCTGCCATAAAA-CGAAGATT-TTTCTTCA----CTT----ATTTCAAGGTTGACCTCGGATCAGGTA : 512
KC477228.1   : CCTCT-GTAGCCGGGTCTGGTTTT-CCAGCCAAAAAA-------------------------------------------------------- : 469
KX037428.1   : CCTCTGTAAGCCGGGTCTGGTCTTTCCTGCCGTAAAA-CGAAGAGA-ACACACCTTGT-CTT-----CTCTATGGTTGACCTCGGATCAGGTA : 519
KU683913.1   : CCTCTGTAAGTCAGGTCTGGTCTTTCCTGCCGTAAAA-CGAAGAGA-ACACACCTTGTTCTTTTTACCTCTA-GGTTGACCTCGGATCAGGTA : 523
AF201704.1   : CCTGTAGC-TT-GGACCCGGTCC--CCTGCCGTAAAA-CA-----------CCCCAAATCT--------TCTAGGTTGACCTCGGATCAGGTA : 547
DQ641634.1   : CCTGTGGTATA-GGTCT--ATCT--CTTGCCGTAAAA-------------------------------------------------------- : 496
EF026121.1   : CCTGTAG--TT-GGACC-GGTCC--CCTGCCGTAAAA-CA-----------CCCCAATTCT--------AAAAGGTTGACCTCGGATCAGGTA : 542
EF026122.1   : CCTGTAG--TT-GGACC-GGTTT--CTTGCCGTAAAA-CA-----------CCCCAATTTTTTT-----TAAAGGTTGACCTCGGATCAGGTA : 541
GU292818.1   : CCCGCGG--TC-GGACC-GGTCC--CCTGCCGTAAAA-CA-----------CACC-ATTTTTT--------AAGGTTGACCTCGGATCAGGTA : 562
GU292822.2   : CTTGT-GAGTT-AGGCC-GGTCC--CCAGCCGTAAAA-C------------CCCCTAATTTT-------TAAAGGTTGACCTCGGATCAGGTA : 539
JQ759872.1   : CCTGTAGC-TT-GGACCCGGTCC--CCTGCCGTAAAA-CA--------C--CCCCCAATTTTA------T--AGGTTGACCTCGGATCAGGTA : 527
JQ760608.1   : CCTATGGT-TT-GGACCCGG-CC--CCTGCCGTAAAA-CAAAAAAA-ACGACACCTAGTGTCTG-----TCAAGGTTGACCTCGGATCAGGTA : 512
JQ846087.1   : CCTGTGGTATA-GGTCT--ATCT--CTTGCCGTAAAA----------ATGCCCTAAAAAAGCAC-----TATAGGTTGACCTCGGATCAGGTA : 557
KC708575.1   : CCTGTAG--TT-GGACC-GGTCC--CCTGCCGTAAAA-CA-----------CCCCAATTCT--------AAAAGGTTGACCTCGGATCAGGTA : 485
KC845930.1   : CCTGTAG--TT-GGACC-GGTCC--CCTGCCGTAAAA-CA-----------CCCCAATTCT--------AAAAGGTTGACCTCGGATCAGGTA : 483
KP133215.1   : CCTGTAGTGCT-GGACC-GGTCC--CCTGCCGTAAAA-CA-----------CCCCCTATTTCT------AAAAGGTTGACCTCGGATCAGGTA : 561
KP133218.1   : CCCGCGG--TC-GGACC-GGTCC--CCTGCCGTAAAA-CA-----------CACC-ATTTTCTC-----GCAAGGTTGACCTCGGATCAGGTA : 565
KU683765.1   : CCTGTAGC-TT-GGACCCGGTCC--CCTGCCGTAAAA-CA--------C--CCCCCAATTTTA------T--AGGTTGACCTCGGATCAGGTA : 527
KU683999.1   : CCTATAG--TT-GGACC-GGTCC--CCTGCCGTAAAA-CG--------C--CCC--AGTATCT------AAAAGGTTGACCTCGAATCAGGTA : 501
KU747773.1   : CCTGCGGT-GC-GTGCCCGGCCC--CCTGCCGTAAAA-C---------C--CCCCCAATTTCT------TTCAGGTTGACCTCGGATCAGGTA : 526
KU747908.1   : CCTGTAGC-CT-AAACCCGGCCC--CCTGCCGTAAAA-CA--------C--AACG-AATATTG------TATAGGTTGACCTCGGATCAGGTA : 500
KX774096.1   : CCTATAG--TT-GGACC-GGTCC--CCTGCCGTAAAA-CG-----------CCCCAGTATTT-------AAAAGGTTGACCTCGAATCAGGTA : 484
KX778681.1   : CCTATAG--TT-GGACC-GGTCC--CCTGCCGTAAAA-CG-----------CCCC-AGTATTT------AAAAGGTTGACCTCGAATCAGGTA : 491
KY250390.1   : CCTGTAG--CT-GGACC-GGTCC--CCTGCCGTAAAA-CA-----------CCCC--AATTTT------TAAAGGTTGACCTCGGATCAGGTA : 544
KY250391.1   : CCTGTAG--CT-GGACC-GGTCC--CCTGCCGTAAAA-CA-----------CCCC--AATTTT------TAAAGGTTGACCTCGGATCAGGTA : 540
LN714575.1   : CCTATAG--TT-GGACC-GGTCC--CCTGCCGTAAAA-CG--------C--CCC--AGTATCT------AAAAGGTTGACCTCGAATCAGGTA : 517
MF153390.1   : CCTGTAG--TT-GGACC-GGTCC--CCTGCCGTAAAA-CA-----------CCCCAATTCT--------GAAAGGTTGACCTCGGATCAGGTA : 510
MF663581.1   : CCTGTGG--TC-GGGCT-AGTCC--CCTGCCGTAAAA-C------------CCCCCAGACTTTT-----TAGTGTTTGACCTCGGATCAGGTA : 532
MG543928.1   : CCTGTAGC-GC-GGGCC-GGTCC--CCTGCCGTAAAA-CA-----------CCCCAATTTT--------TATAGGTTGACCTCGGATCAGGTA : 506
MG543932.1   : CCTGTAGC-GT-GAGCC-GGTCC--CCTGCCGTAAAA-CA-----------CCCCAATTTT--------TATAGGTTGACCTCGGATCAGGTA : 507
MG543935.1   : CCTGTAGC-TT-GGACCCGGTCC--CCTGCCGTAAAA-CA-----------CCCCAAATCT--------TCTAGGTTGACCTCGGATCAGGTA : 507
MG543943.1   : CCTGTAGC-GC-GGGCC-GGTCC--CCTGCCGTAAAA-CA-----------CCCCAATTTT--------TATAGGTTGACCTCGGATCAGGTA : 507
MG543947.1   : CCTGTAGC-GC-GGGCC-GGTCC--CCTGCCGTAAAA-CA-----------CCCCAATTTT--------TATAGGTTGACCTCGGATCAGGTA : 507
MG832444.1   : CCTACAG--TT-GGACC-GGTCC--CCTGCCGTAAAA-CG-----------CCCC-AGTCTTT------AAAAGGTTGACCTCGGATCAGGTA : 525
MH178723.1   : CCTGTAGC-GC-GGGCC-GGTCC--CCTGCCGTAAAA-A------------CCCCAAATTCT-------TATAGGTTGACCTCGGA------- : 518
MH305505.1   : CCCGCGG--TC-GGACC-GGTCC--CCTGCCGTAAAA-CA-----------CACC-ATTTTTT--------AAGGTTGACCTCGGATCAGGTA : 494
MH931277.1   : CCTGTAGC-TT-GGACCCGGTCC--CCTGCCGTAAAA-CA--------C--CCCCCAATTT--------TATAGGTTGACCTCGGATCAGGTA : 548
MK192915.1   : CCTGTAGCCCA-AG-CCCGGCGC--CCTGCCGTAAAA-CAC----------CCCCCAGTGT---------ATAGGTTGACCTCGGATCAGGTA : 488
MK367486.1   : CCTATAG--TT-GGACC-GGTCC--CCTGCCGTAAAA-CG-----------CCCC-AGTATTT------AAAAGGTTGACCTCGAATCAGGTA : 512
MK367543.1   : CCTGTAGC-GC-GGGCC-GGTCC--CCTGCCGTAAAA-CA-----------CCCCAATTCT--------TATAGGTTGACCTCGGATCAGGTA : 524
NR_160210.1  : CCTGTAGC--C-GGGCCCGGTGC--CCTGCCGTAAAA-CA-----------CACCTATTTTC-------TCAAGGTTGACCTCGGATCAGGTA : 543
```

Figure S19: ITS sequence alignment of UM10M with sequences published in GenBank

```
                      660         *        680         *
UM10M       : GGAATACCCGCTGAACTTAAGCATATCAATAAG-CGGAGGA : 578
JQ761479.1  : GGAATACCCGCTGAACTTAAGCATATCAATAAG-CGGAGGA : 552
KC477228.1  : ----------------------------------------- :   -
KX037428.1  : GGAATACCCGCTGAACTTAAGCATATCAATAAG-CGGAGGA : 559
KU683913.1  : GGAATACCCGCTGAACTTAAGCATATCAATAAG-CGGAGGA : 563
AF201704.1  : GGAATACCCGCTGAACTTAA--------------------- : 567
DQ641634.1  : ----------------------------------------- :   -
EF026121.1  : GGAATACCCGCTGAACTTAAGCATATCAATAAG-CGGAGGA : 582
EF026122.1  : GGAATACCCGCTGAACTTAAGCATATCAATAAG-CGGAGGA : 581
GU292818.1  : GGGATACCCGCTGAACTTAAGCATATCAATAAGC-GGAGGA : 602
GU292822.2  : GGAATACCCGCTGAACTTAAGCATATCAATAAG-CGGAGGA : 579
JQ759872.1  : GGAATACCCGCTGAACTTAAGCATATCAATAAG-CGGAGGA : 567
JQ760608.1  : GGAATACCCGCTGAACTTAAGCATATCAATAAG-CGGAGGA : 552
JQ846087.1  : GGAATACCCGCTGAACTTAAGCATATCAT------------ : 586
KC708575.1  : GGAATACCCGCTGAACTTAAGCATATCAA------------ : 514
KC845930.1  : GGAATACCCGCTGAACTTAAGCATATCA------------- : 511
KP133215.1  : GGAATACCCGCTGAACTTAAGCATATCAATAAG-CGGAGGA : 601
KP133218.1  : GGGATACCCGCTGAACTTAAGCATATCAATAAGC-GGAGGA : 605
KU683765.1  : GGAATACCCGCTGAACTTAAGCATATCAATAAG-CGGAGGA : 567
KU683999.1  : GGAATACCCGCTGAACTTAAGCATATCAATAAG-CGGAGGA : 541
KU747773.1  : GGAATACCCGCTGAACTTAAGCATATCAATAAG-CGGAGGA : 566
KU747908.1  : GGAATACCCGCTGAACTTAAGCATATCAATAAG-CGGAGGA : 540
KX774096.1  : GGAATACCCGCTGAACTTAA--------------------- : 504
KX778681.1  : GGAATACCCGCTGAACTTAAGCATATCAATAAG-CGGAGGA : 531
KY250390.1  : GGAATACCCGCTGAACTTAAGCATATCA-TA---------- : 574
KY250391.1  : GGAATACCCGCTGAACTTAAGCATATCAATAAG-------- : 573
LN714575.1  : GGAATACCCGCTGAACTTAAGCATATCAATAAG-CGGAGGA : 557
MF153390.1  : GGAATACCCGCTGAACTTAAGCATATCAATAAG-CGGAGGA : 550
MF663581.1  : GGGATACCCGCTGAACTTAA--------------------- : 552
MG543928.1  : GGAATACCCGCTGAACTTAA--------------------- : 526
MG543932.1  : GGAATACCCGCTGAACTTAA--------------------- : 527
MG543935.1  : GGAATACCCGCTGAACTTAA--------------------- : 527
MG543943.1  : GGAATACCCGCTGAACTTAA--------------------- : 527
MG543947.1  : GGAATACCCGCTGAACTTAA--------------------- : 527
MG832444.1  : GGAATACCCGCTGAACTTAAGCAT----------------- : 549
MH178723.1  : ----------------------------------------- :   -
MH305505.1  : GGGATACCCGCTGAACTTAAGCATAT--ATAAGCCGGAGGA : 533
MH931277.1  : GGAATACCCGCTGAACTTAAGCATATCAATAAG-CGGAGGA : 588
MK192915.1  : GGAATACCCGCTGAACTTAAGCATATCAATAAG-CGGAGGA : 528
MK367486.1  : GGAATACCCGCTGAACTTAAGCATATCAATAAGGCGGAGGA : 553
MK367543.1  : GGAATACCCGCTGAACTTAA--------------------- : 544
NR_160210.1 : GGAATACCCGCTGAACTTAAGCATATCAATA---------- : 574
```

Table S1: Sequences used for alignment analysis and to identify close relatives

UM10M	
JQ761479.1*	*Nemania* sp. genotype 547 isolate NC0453
KU683913.1*	*Nemania* sp. ARIZ FL1860
KX037428.1*	*Nemania* sp. strain BCC30850
AF201704.1	*Nemania aenea* variety *aureolatum*
DQ641634.1	*Nemania plumbea*
EF026121.1	*Nemania primolutea* isolate 91102001
EF026122.1	*Nemania illita* isolate 236
GU292818.1	*Nemania bipapillata* isolate 90080610
GU292822.2	*Nemania maritima* isolate 89120401
JQ759872.1	*Nemania serpens* isolate AK1587
JQ760608.1	*Nemania beaumontii* isolate FL0980
JQ846087.1	*Nemania plumbea* isolate 6540
KC477228.1	*Nemania pouzarii* strain ATCC 2612
KC708575.1	*Nemania primolutea* strain NY2
KC845930.1	*Nemania primolutea* isolate FG9
KP133215.1	*Nemania abortiva* isolate 18.2
KP133218.1	*Nemania bipapillata* isolate 64.2
KU683765.1	*Nemania serpens* isolate CBS 679.86
KU683999.1	*Nemania diffusa* isolate NC1333
KU747773.1	*Nemania* sp. strain F1454
KU747908.1	*Nemania* sp. strain F2130
KX774096.1	*Nemania* sp. strain 43
KX778681.1	*Nemania diffusa* isolate CNUZ-L414
KY250390.1	*Nemania* sp. voucher GAB127
KY250391.1	*Nemania* sp. voucher GAB137
LN714575.1	*Nemania diffusa*
MF153390.1	*Nemania primolutea* isolate ZCL5
MF663581.1	*Nemania* sp. strain LTL428
MG543928.1	*Nemania* sp. isolate EP04
MG543932.1	*Nemania* sp. isolate EP20
MG543935.1	*Nemania* sp. isolate EP26
MG543943.1	*Nemania* sp. isolate PA19
MG543947.1	*Nemania* sp. isolate PA33
MG832444.1	*Nemania* sp. strain TS-24
MH178723.1	*Nemania* sp. isolate IUB:B.Whitaker:OTU54
MH305505.1	*Nemania bipapillata* strain Ct-BC70
MH931277.1	*Nemania serpens* isolate m22
MK192915.1	*Nemania* sp. isolate S1.5
MK367486.1	*Nemania* sp. isolate Z-Y-43
MK367543.1	*Nemania* sp. isolate Z-G-12
NR_160210.1	*Nemania macrocarpa* CBS 109567

*) BLAST hit
